# Electrochemical Removal of BSA Foulant Using a Stabilised Conductive PVDF Membrane Incorporated with Multiwall Carbon Nanotubes

**DOI:** 10.3390/membranes16100351

**Published:** 2026-10-21

**Authors:** Zihao Guan, Andrew J. Cruden

**Affiliations:** Department of Mechanical Engineering, University of Southampton, Highfield Campus, Southampton SO17 1BJ, UK; a.j.cruden@soton.ac.uk

**Keywords:** PVDF membrane, multiwall carbon nanotubes (CNTs), conductive membrane, electrochemical rinsing, BSA fouling, polarisation

## Abstract

Membrane fouling limits the sustainable application of polymeric membranes in wastewater treatment and water reuse. Strong adsorption of biomacromolecules and microorganisms can cause irreversible fouling, reducing membrane permeability and operational lifetime. This study developed conductive polyvinylidene fluoride (PVDF) membranes with multiwalled carbon nanotubes (hereafter referred to as CNTs) for electrochemically assisted removal of bovine serum albumin (BSA) foulants. A CNT-incorporated membrane (PVDF–CNT), prepared by phase inversion, and a CNT surface-coated membrane (CNT/PVDF), fabricated by vacuum filtration, were compared with pristine PVDF and a commercial hydrophilic PVDF membrane (c-PVDF). PVDF–CNT membrane exhibited an outstanding electrochemical response at approximately +1.6 V and the most pronounced signal variation after BSA addition. This enhanced response might be contributed by the combined contributions of β-phase-associated dipolar polarisation and interfacial polarisation at the CNT and PVDF interface, potentially promoting local charge accumulation and redistribution. After 3 min of electrochemical rinsing at +1.6 V in 0.2 mol/L Na_2_SO_4_ electrolyte, the PVDF–CNT membrane achieved a flux recovery ratio (FRR; the percentage of flux recovered after rinsing) of 67%, exceeding those of the pristine PVDF membrane (55%) and c-PVDF membrane (47%). Although the CNT/PVDF membrane exhibited a higher apparent FRR (91%), this behaviour was attributed to pore blockage by the deposited CNT layer and partial CNT detachment during rinsing, indicating limited structural stability. Across four consecutive fouling–rinsing cycles, the PVDF–CNT membrane showed smaller reductions in flux recovery (42.1%, 4.7%, and 23.8%) than c-PVDF (65.5%, 21.6%, and 25.5%). These findings highlighted the structural stability and electrochemical rinsing performance of the PVDF-CNT membrane and also suggested that tailoring the membrane’s combined polarisation-related behaviours offered a direction for improving electrochemically assisted foulant removal and flux recovery.

## 1. Introduction

Membrane filtration technology using polymeric membranes has been recognised as a leading solution for recovering water from various wastewater streams for reuse because it can remove particles and foulants from water. However, polymer membranes must deal with great challenges from reversible and irreversible membrane fouling during filtration operation, in which the irreversible fouling induced by the tight binding of organic and microorganism particles, especially proteins, on the surfaces of the membrane and pores is extremely detrimental. Effective techniques to resist fouling and remove deposited foulants are highly desirable in the membrane separation field [1,2]. BSA, a widely used model protein foulant, was therefore selected in this study to evaluate membrane fouling and electrochemical rinsing performance.

PVDF composite membranes are widely employed in industry owing to their tensile strength of approximately 40–60 MPa, high ductility, good thermal stability, and broad chemical resistance to most acids, oxidants, alcohols, hydrocarbons, and many organic solvents, while strategies to improve their antifouling and defouling properties have been extensively investigated. It is thought that a promising solution is to incorporate fillers, such as CNTs [3], glass fibres [4], SiO_2_ nanoparticles [5], TiO_2_ nanotubes [6], etc., into the polymer matrix to enhance its mechanical strength, conductivity and hydrophilicity. Among them, CNTs are particularly attractive because they can simultaneously modify multiple membrane properties, including wettability, pore structure, mechanical strength, surface functionality, electrochemical responsiveness and so forth [7,8]. Therefore, CNTs have been widely explored as a versatile nanofiller for PVDF membrane modification.

Previous studies demonstrated that CNT-embedded PVDF membranes could efficiently utilise electrostatic forces to repel foulants and electrochemical reactions (oxidation and reduction) to degrade/remove apparently irreversible foulants [9]. Hou et al. fabricated a CNTs-PVDF hollow fibre membrane by surface loading acidified CNTs onto a commercial PVDF hollow fibre membrane and stabilising the CNT coating through PVA/SA cross-linking. During filtration, an applied bias was used to mitigate fouling, with −1.2 V identified as the optimal voltage. After subsequent hydraulic rinsing, the membrane permeance recovered to 95.39% of its initial value, compared with only 57.98% for the membrane operated without electrical assistance [10]. Vacuum-filtration type coating was used as a facile method to deposit CNT directly onto polymer membranes for electrical field-assisted crossflow filtration, realising excellent conductivity and antifouling performance [11], though such surface-coated membranes brought CNT leakage risks under tangential-flow operation due to shearing force, hence inappropriate to be used in dead-end filtration when back-washing/rinsing is needed. Alternatively, the CNTs can be blended with polymer(s) in the casting solution to form CNT-doped composite membranes, allowing both dead-end and crossflow filtration operation [12]. Zhang et al. fabricated a CNT–PVDF conductive ultrafiltration membrane by a two-step phase inversion method. During surface water filtration, the membrane operated at an optimal voltage of −2.5 V and exhibited a 41.6% reduction in irreversible fouling growth rate, a 49.9% decrease in irreversible fouling resistance, and a 31.7% higher flux recovery rate than the membrane operated without power [13].

CNTs can also induce the formation of β-phase over α-phase PVDF when incorporated into the PVDF matrix, leading to the coexistence of both phases [14]. The β phase of PVDF is widely recognised as the electroactive phase due to its all-trans planar zigzag chain conformation, which produces a strong net dipole moment. The alignment of these dipoles leads to large spontaneous polarisation, thereby giving rise to its piezoelectric, pyroelectric, and ferroelectric properties [15]. In a recent study, Zhang et al. fabricated CNTs–PVDF composite membranes by wet-spinning, where CNT incorporation promoted polar β-phase PVDF formation and strengthened CNT–PVDF interfacial coupling, thereby facilitating charge redistribution and electron transport within the membrane. Under −1.35 V vs Ag/AgCl, the membrane exhibited markedly improved antifouling behaviour, retaining ~95% of its initial flux after 2 h of humic acid filtration and extending the backwash interval by 12.6–19 times compared with commercial PVDF membranes, with nearly complete flux recovery after backwashing [16]. Furthermore, the large difference in electrical conductivity between PVDF and CNTs can induce interfacial (Maxwell–Wagner–Sillars) polarisation at the CNT and PVDF interface [17]. The coexistence of electroactive β-phase PVDF and interfacial polarisation at the CNT and PVDF interface is expected to further enhance charge accumulation and redistribution within the membrane matrix, potentially boosting the electrochemical foulant removal [18,19].

In this work, three types of PVDF-based membranes were fabricated, including pristine PVDF membranes, PVDF-CNT conductive composite membranes prepared via a phase inversion method, and CNT/PVDF membranes fabricated by vacuum filtration. A c-PVDF membrane was also included as a benchmark for systematic filtration performance comparison. This study clarified how different CNT integration strategies influence membrane properties, electrochemical responsiveness and filtration performance. In particular, electrochemical characterisation was employed to identify the active electrochemical response of membranes and to guide the selection of a rational electrochemical rinsing potential. By combining membrane characterisation, filtration tests, comparative electrochemical analysis, and fluorescein isothiocyanate–conjugated bovine serum albumin (FITC-BSA) visualisation, this work further explored whether incorporating CNTs into a PVDF matrix could provide a more stable and effective route for fouling control and flux recovery than surface coating, while also offering insight into the possible contribution of polarisation-related behaviours in membrane electrochemical rinsing.

## 2. Materials and Methods

### 2.1. Materials and Chemicals

Polyvinylidene fluoride (PVDF, average molecular weight, Mw = 534,000 g/mol by GPC) powder, 1-methyl-2-pyrrolidinone (NMP, 99.5%), ethanol (EtOH, anhydrous, ≥99.5%), bovine serum albumin (BSA, purity ≥ 98%), anhydrous sodium sulphate (Na_2_SO_4_, 99%), and fluorescein isothiocyanate–conjugated bovine serum albumin (FITC-BSA) were purchased from Merck (Gillingham, UK).Multi-walled carbon nanotubes (MWCNTs, 95% purity, specific surface area > 220 m^2^/g) were purchased from Ossila (Sheffield, UK), with internal diameter, outer diameter, and lengths of 5–10 nm, 8–18 nm, and 10–30 µm, respectively. De-ionised (DI) water was collected from the MilliQ water purification system (Millipore SAS, Molsheim, France). Polyvinylpyrrolidone K-30 (PVP-K30) was obtained from Fluka (Buchs, Switzerland). Commercial hydrophilic PVDF membrane (pore size: 0.1 µm) was purchased from Delvslab, Jiaxing, China.

### 2.2. Membrane Fabrication

#### 2.2.1. PVDF Membrane

The PVDF membrane samples were fabricated via the thermally non-solvent induced phase separation (T-NIPS) method, in which the PVDF, PVP, EtOH, NMP, and DI water served as the polymer, additive, conductive carbon filler, co-solvent, solvent, and non-solvent, respectively. The casting solution was made by initially dispersing the PVP in the NMP-EtOH solution, followed by water bath sonication for 30 min and then the addition of PVDF powder. The calculated quantities of each material were listed in Table 1. The mixture was then heated (80 degrees) and stirred for 4 h. Once the solution became homogeneous and a visually uniform dispersion was obtained, with no visible aggregates (e.g., PVDF white aggregation), it was set aside overnight to allow the removal of the bubbles in the solution.

Before casting the membrane, the cooled casting solution needed to be heated to 60 degrees for 20 min to achieve a viscosity that was appropriate to be cast. The heated solution was poured onto the glass, which was cleaned with EtOH, and then heated for 5 min in the AFA-III automatic thick film coater (MTI Co.), which was set at 80 degrees. After being cast by the casting knife (200 µm), the non-solid-state membrane together with the glass plates needed to be cooled down for 40 s in the air before immersing in DI water to form a solid membrane.

#### 2.2.2. PVDF-CNT Membrane

The PVDF-CNT membrane was fabricated using the same procedure as that of the PVDF membrane, except that the casting solution was made by dispersing the CNT in the NMP-EtOH-PVP solution by water bath sonication for 30 min, followed by the addition of PVDF powder [20,21,22]. The calculated quantities of each material were listed in Table 1.

#### 2.2.3. CNT/PVDF Membrane

A CNT/PVDF membrane was prepared using the vacuum filtration method. First, a uniform CNT suspension was prepared by dispersing CNT in 30 mL DI water with ultrasonication for 30 min to ensure homogeneous dispersion. The CNT coating mass per unit membrane area was 0.07251 mg/cm^2^ (The calculation was provided in Supplementary Materials S1. The PVDF membrane was then placed on a filter, and the CNT suspension was slowly filtered through under vacuum. During filtration, CNTs deposited evenly on the membrane surface, forming a thin conductive layer. After filtration, the composite membrane was slightly rinsed with deionised water to remove residual impurities and dried at room temperature (20–24 °C) for 24 h to obtain the CNT-PVDF membrane.

### 2.3. Filtration Performance

The filtration experiments were conducted in a vacuum-driven dead-end configuration at room temperature (20–24 °C). A 500 mL feed conical flask was used, and the feed solution was replenished as needed. The feed side was open to atmospheric pressure, while the conical flask on the permeate side was evacuated using a diaphragm vacuum pump to maintain a constant vacuum. ((FB65499, WELCH-ILMVAC/ILMVAC GmbH, Ilmenau, Germany; ultimate pressure <100 mbar; pumping speed 15 L min^−1^). The same pump setting and filtration configuration were used for all membrane samples. The filtration set-up was illustrated in Supplementary Materials Figure S1. Prior to filtration, the membrane was immersed in DI water for 30 min to ensure complete wetting, after which the filtration experiment was immediately started. DI water was the feed solution of the first stage (J_w0_) (Unit: Litre per Square Metre per Hour (LMH)); 2 g/L BSA contamination (PH = 6.6) was the second stage. BSA filtration continued until the flux became stable, and the first 35 min of BSA fouling stages were used; after contamination, DI water would act as the feed solution to quantify the extent of contamination, which was the third step (J_w1_). The fourth step also used DI water as the feed solution after the fouled membrane was rinsed in 0.2 mol/L Na_2_SO_4_ with or without +1.6 V for 3 min (J_w2_). Besides the fouling stage, the common analysis duration of each remaining pure water (PW, refers to DI water) filtration stage was also the same for membrane samples. The rinsing set-up was illustrated in Supplementary Materials Figure S2. After completing the filtration test, the flux recovery ratio (FRR %), total fouling ratio (R_t_ %) and rinsing recovery ratio (R_C_ %) were calculated from the flux value at the end of each filtration stage to assess the BSA removal performance of the membranes (When the flux fluctuated repeatedly at the end period, the mean value was used), using the following equations could be obtained:

(1) $$\mathrm{FRR}=\frac{J_{w2}}{J_{w0}}\;\times \;100\%$$

(2) $$R_{t}=(1-\frac{J_{w1}}{J_{w0}})\times 100\%$$

(3) $$R_{C}=\frac{\left(J_{w2}-J_{w1}\right)}{J_{w0}}\times 100\%$$

The flux data were smoothed using locally weighted scatterplot smoothing (LOWESS) in Origin 2025b (OriginLab Corporation, Northampton, MA, USA) to visualise the overall temporal trend. A smoothing span of 0.20 was applied, while all original experimental data points were retained.

### 2.4. Characterisations

Water contact angle (WCA) is the commonly utilised method in laboratories to determine the wettability of the membrane. WCAs were measured using a drop shape analyser (DSA-100, Kruss, Hamburg, Germany) by the sessile-drop method. Scanning electron microscopy (SEM) (JEOL JSM-7200F, Akishima, Tokyo, Japan) and energy-dispersive X-ray spectroscopy (EDS) (Oxford Instruments, High Wycombe, UK) was applied to observe morphology and elements after the membrane samples were sputter-coated with Au for 60 s. X-ray diffraction (XRD) (D2 PHASER, Bruker AXS, Karlsruhe, Germany) analysis was carried out using a Cu Kα radiation source (λ = 1.54184 Å) operated at 30 kV and 10 mA. The diffraction patterns were recorded in locked-coupled scan mode over a 2θ range of 5.07–60.00° with a step size of 0.0203° and a counting time of 38.4 s per step. A divergence slit of 1 mm was used. Raman spectroscopy was performed using a Renishaw in Via Raman microscope (Wotton-under-Edge, UK) to analyse the vibrational modes of the membranes, providing insight into their crystal structure and molecular backbone characteristics. The measurements were carried out at 0.05% laser power with an exposure time of 60 s.

### 2.5. Electrochemical Tests

The electrochemical characterisations were performed on the electrochemical workstation (Autolab PGSTAT302n, Metrohm, The Netherlands). The membrane, Pt electrode, and Ag/AgCl electrode were used as the working electrode (WE), counter electrode (CE), and reference electrode (RE), respectively. All the membranes for the electrochemical tests had the same area exposed to the electrolyte. The cyclic voltammetry (CV) curves of all the membranes in 0.2 mol/L Na_2_SO_4_ and 0.2 mol/L Na_2_SO_4_ + 2 g/L BSA were also measured at a scan rate of 100 mV/s in order to study the membrane electrochemical interaction with BSA. Linear sweep voltammetry (LSV) curves were measured in 0.2 mol/L Na_2_SO_4_ solution with a scan rate of 1 mV/s to characterise the oxygen evolution potential (OEP) as well as the electrochemical kinetics of all the membrane samples around a certain potential range. Electrochemical impedance spectroscopy (EIS) was performed under open-circuit potential (OCP) for all the CNT-containing membranes to measure the wet membrane resistance, encompassing a frequency range from 800 kHz down to 0.1 Hz. Moreover, electrochemical tests were also performed on the PVDF-CNT membrane during each filtration stage to verify the effectiveness of BSA removal.

### 2.6. Stability Test

The stability tests included filtration stability tests and electrochemical stability tests. In addition to the stages described in 2.3, the membrane was further fouled using a 2 g/L BSA solution, followed by electrochemical rinsing in Na_2_SO_4_ electrolyte at an applied potential of +1.6 V and subsequent PW filtration. This fouling–rinsing–filtration cycle was repeated three times. Including the rinsing cycle at 2.3, a total of four fouling–electrochemical rinsing cycles were performed.

CV was carried out in 0.2 mol/L Na_2_SO_4_ over the potential range 0 to 2 V vs Ag/AgCl at a scan rate of 100 mV/s to evaluate the electrochemical stability. The CNT-related membranes were subjected to 50 consecutive CV cycles under identical conditions. CV curves from selected cycles (2, 50) were compared.

### 2.7. Electrochemical Rinsing Visualisation

Electrochemical rinsing visualisation tests were performed on PVDF-CNT and c-PVDF membranes. Prior to fouling, photographs of the clean membranes were taken, and the PW flux was measured. The membranes were then fouled using a solution containing 2 g/L BSA and 50 mg FITC-BSA. After fouling, the membranes were removed, dried, and photographed. Electrochemical rinsing was then conducted at +1.6 V for 3 min, after which the PW flux was measured again. All fluorescence images were taken in the dark environment using blue-light excitation, with the emitted fluorescence observed through orange laboratory goggles.

## 3. Results and Discussions

### 3.1. Membrane Characterisations

#### 3.1.1. Wettability

**Figure 1:**
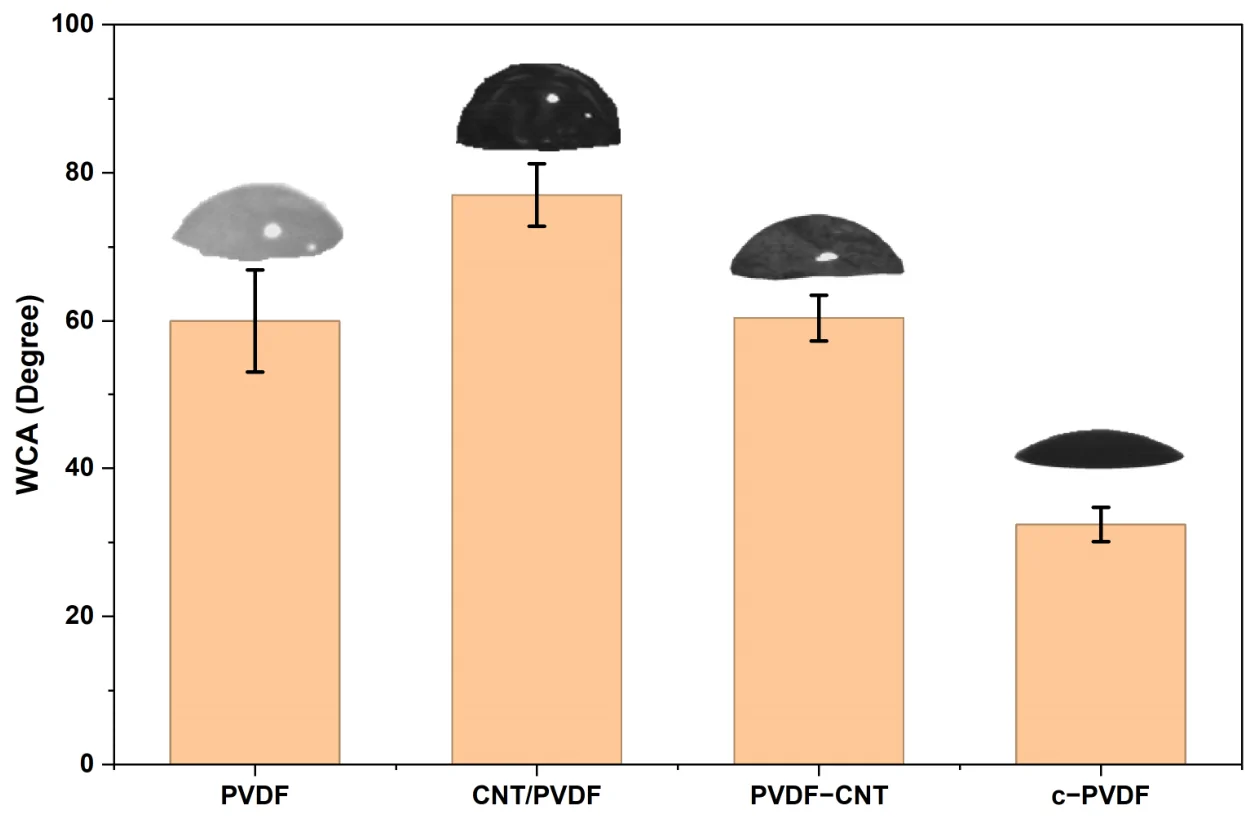
The WCAs of PVDF, CNT/PVDF, PVDF-CNT and c-PVDF membranes.

The pristine PVDF membrane exhibited a relatively high WCA (60 ± 6.9°), indicating its inherent hydrophobic nature, whereas the c-PVDF membrane achieved the lowest WCA (32.4 ± 2.3°) due to the hydrophilic surface modification. After CNT incorporation, the WCA did not change notably (60.4 ± 3.1°), since the CNT network was covered by the PVDF (Figure 1). In contrast, the CNT/PVDF membrane showed an evident increase in WCA (77 ± 4.2°), likely because the CNT layer was more inherently hydrophobic than PVDF [23].

#### 3.1.2. SEM

**Figure 2:**
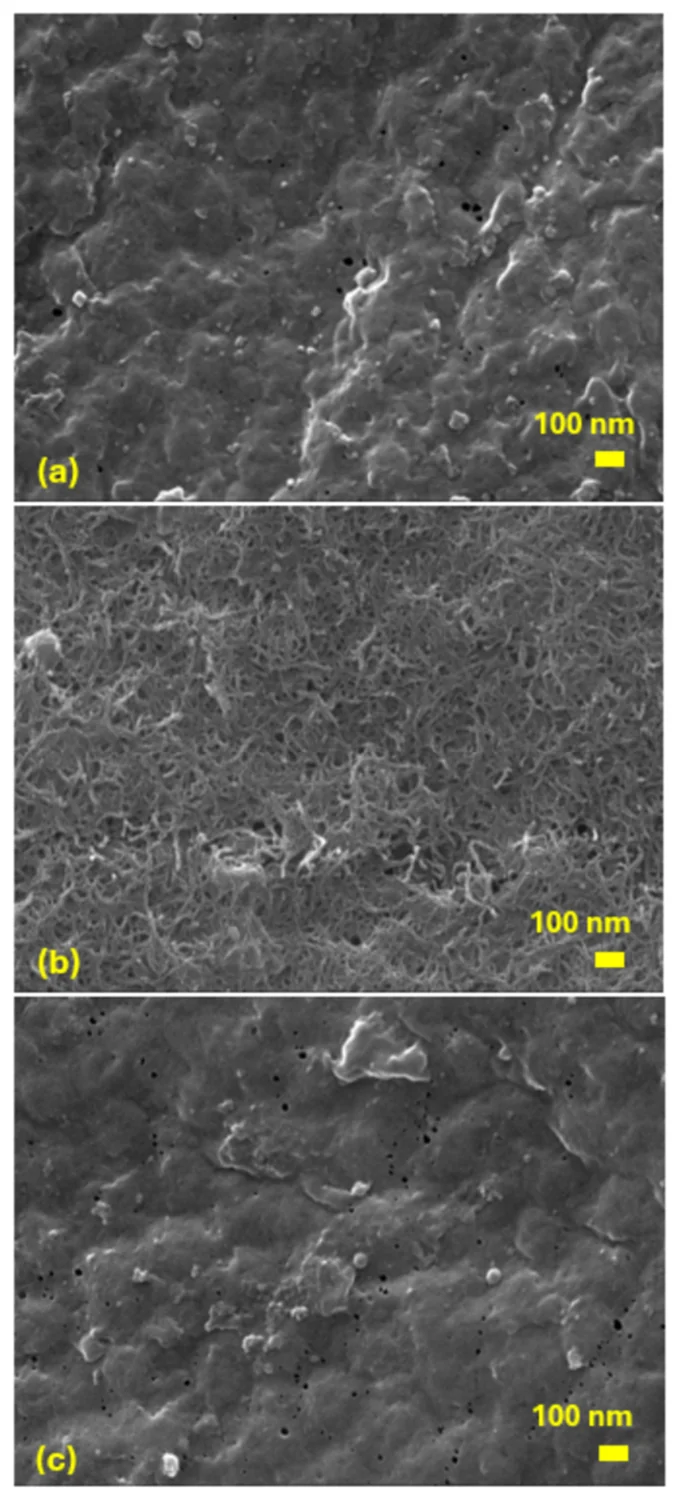
The SEMs of PVDF (**a**), CNT/PVDF (**b**), and PVDF-CNT (**c**) membranes showing morphologies of each membrane.

Within the selected membrane surface regions shown in Figure 2, the apparent pore sizes were estimated to range from approximately 1 to 50 nm. The pristine PVDF and PVDF-CNT membranes showed a relatively smooth surface with uniformly distributed pores (Figure 2a,c). After CNT incorporation, the membrane surface did not exhibit obvious variations, with CNTs well integrated within the polymer matrix, indicating strong interfacial interaction and uniform dispersion (Figure 2c). In contrast, the CNT/PVDF membrane exhibited a thin CNT layer attached to the membrane surface, where agglomeration of CNTs could be observed (Figure 2b). Furthermore, the CNT surface coating layer would inevitably block the membrane pores, leading to a decrease in permeate flux.

#### 3.1.3. EDS

**Figure 3:**
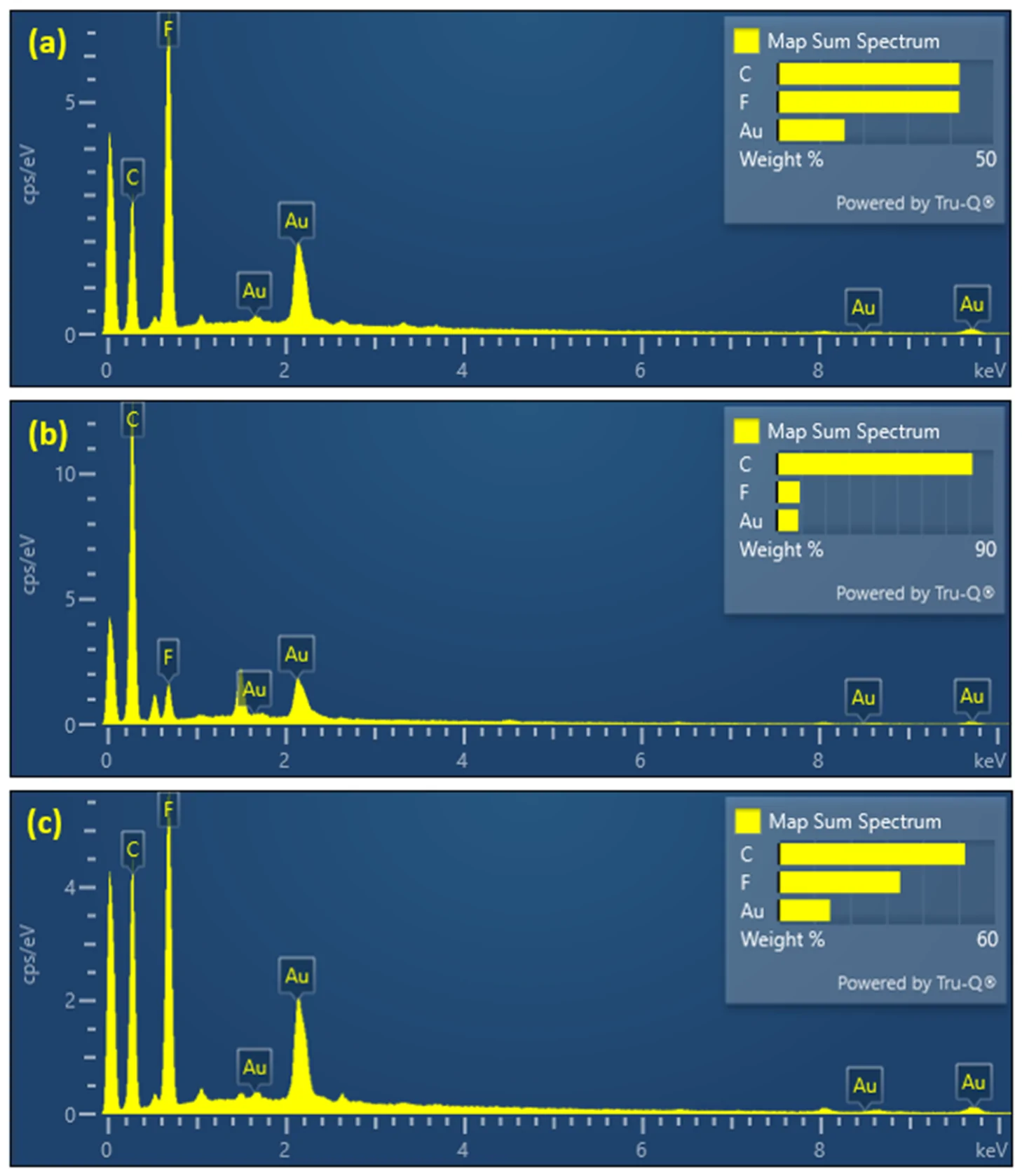
The EDSs of PVDF (**a**), CNT/PVDF (**b**) and PVDF-CNT (**c**) membranes.

EDS at 15 kV revealed characteristic C and F peaks in pristine PVDF (Figure 3a). PVDF-CNT membrane moderately increased the C signal (Figure 3c), although surrounding PVDF attenuated low-energy C Kα X-rays from embedded CNTs. CNT/PVDF membrane produced a stronger C signal and a lower relative F signal (Figure 3b), consistent with surface CNT enrichment. These qualitative to semi-quantitative results reflect local elemental composition rather than absolute CNT content. A thin gold layer was sputter-coated onto the membranes to improve conductivity and signal stability.

#### 3.1.4. XRD

**Figure 4:**
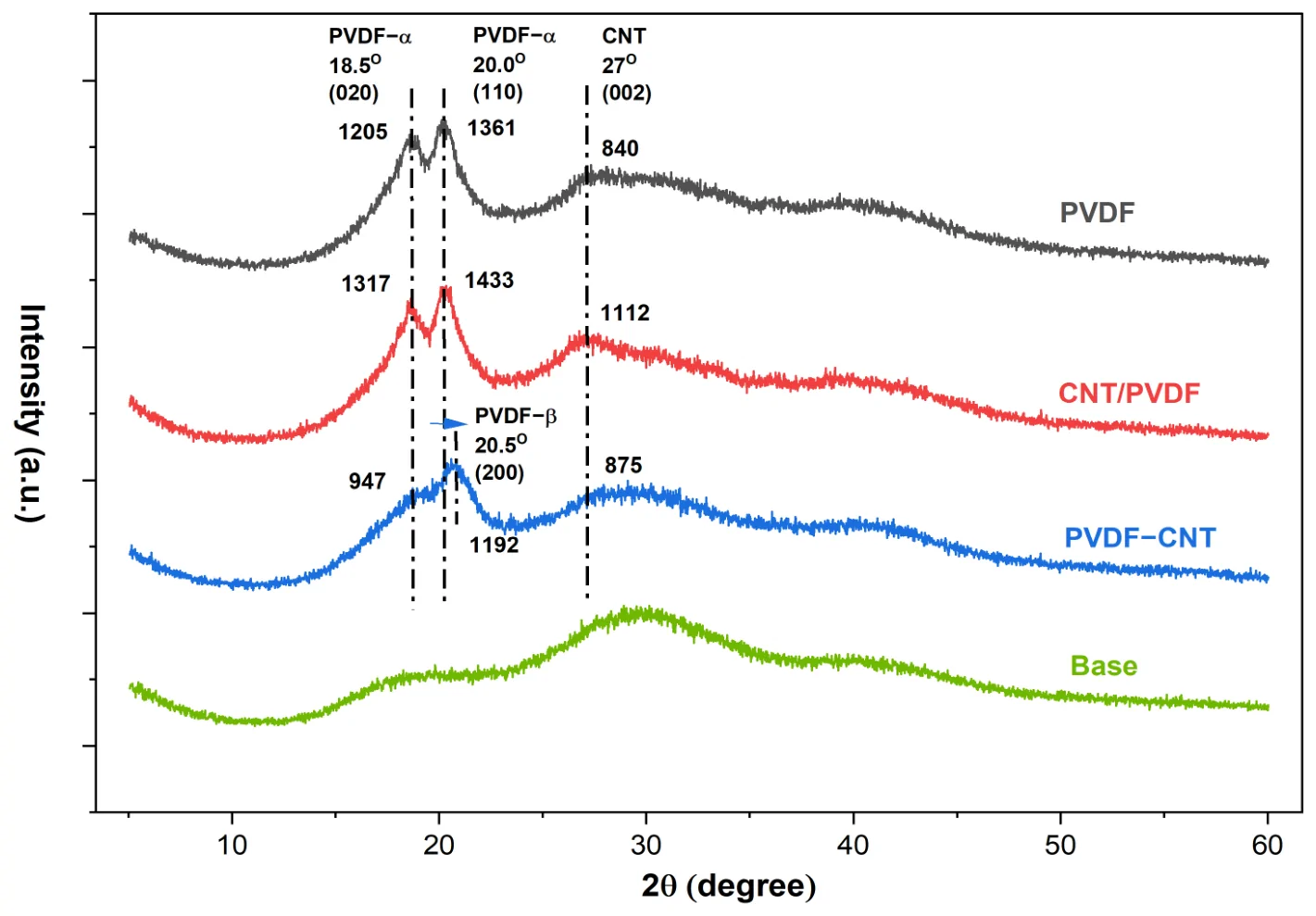
The XRDs of PVDF, CNT/PVDF, and PVDF-CNT membranes. The number near the peaks represents peak intensity.

The pristine PVDF membrane exhibited characteristic diffraction peaks corresponding to the α (020) and (110) crystalline phases of PVDF [24]. The CNT/PVDF membrane showed similar PVDF peaks with additional weak diffraction signals corresponding to the graphitic structure of CNTs (002), confirming their presence on the membrane surface [25]. After CNT incorporation, the overall crystallinity slightly decreased, and the relative PVDF peak position of the α-phase (110) peak slightly shifted to 20.5 degrees, suggesting that CNTs acted as nucleating agents promoting the electroactive β-phase formation [26]. However, the CNT peak was hardly observed, which might be due to the low amount of CNT and the CNT network was covered by the PVDF (Figure 4). These results indicate that CNT incorporation influenced the internal crystalline structure of PVDF, while surface coating primarily contributed to the external addition of CNTs without significant alteration of the polymer matrix. There were some peaks appearing above 30 degrees, which were caused by the sample holder base.

#### 3.1.5. Raman

**Figure 5:**
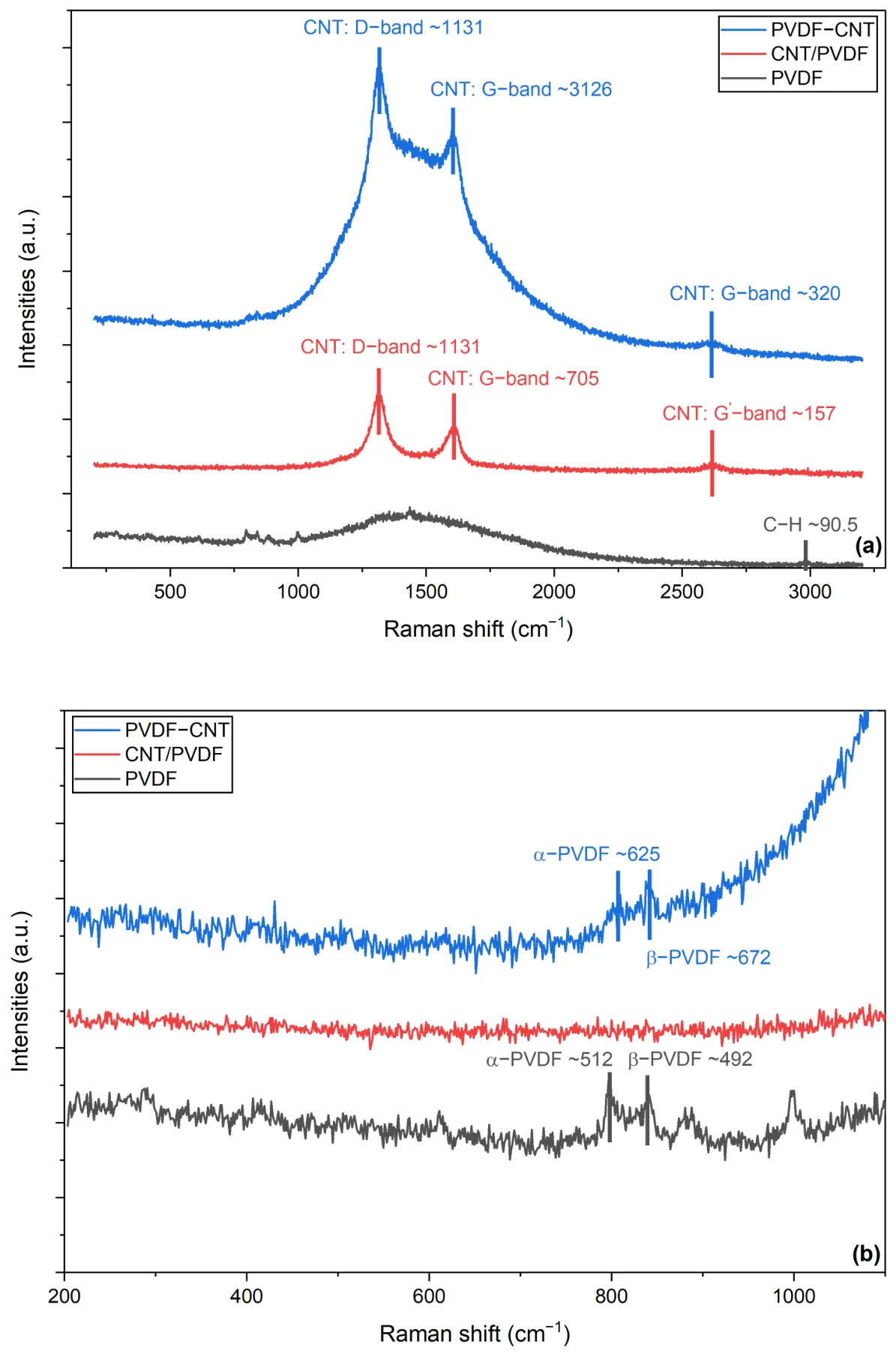
The Raman spectra of PVDF, CNT/PVDF and PVDF-CNT membranes (**a**); the enlarged view of the Raman spectra in the range of 200–1100 cm^−1^ (**b**). The numbers near the peaks represents peak intensity.

The broad Raman feature of pristine PVDF (~1000–2000 cm^−1^) was attributed to the overlap of multiple polymer-chain vibrational modes and the structural disorder associated with the amorphous regions of the semicrystalline PVDF matrix. Three low-intensity bands could be observed around ~800–1000 cm^−1^, which were attributed to α-PVDF, β-PVDF, and C–C skeletal stretching vibrations of the PVDF polymer chain, respectively. In the high-wavenumber region, a very weak feature near ~2900–3000 cm^−1^ was associated with C–H symmetric stretching. Distinct Raman bands corresponding to CNT-related D (~1350 cm^−1^), G (~1580 cm^−1^), or G^’^ (~2600–2700 cm^−1^) modes were observed in PVDF-CNT and CNT/PVDF membranes, confirming the successful incorporation and surface coating of CNT [27,28]. However, CNT-related peaks were much clearer in the surface-coated membrane, and the PVDF-related peaks were almost vanished, indicating CNT covering the membrane surface. Meanwhile, the PVDF-CNT membrane exhibited much broader and higher, yet clearly distinguishable, D and G bands. This feature could be attributed to the overlap of PVDF-related bands and CNT-related bands in the 1000–2000 cm^−1^ region, confirming the successful embedding of CNTs within the PVDF matrix. The intensity of the β-PVDF peak was higher than that of the PVDF membrane, further indicating that the CNTs promote the formation of electroactive β-PVDF (Figure 5). These findings were consistent with the EDS and SEM observations.

### 3.2. Electrochemical Performance of Membranes

**Figure 6:**
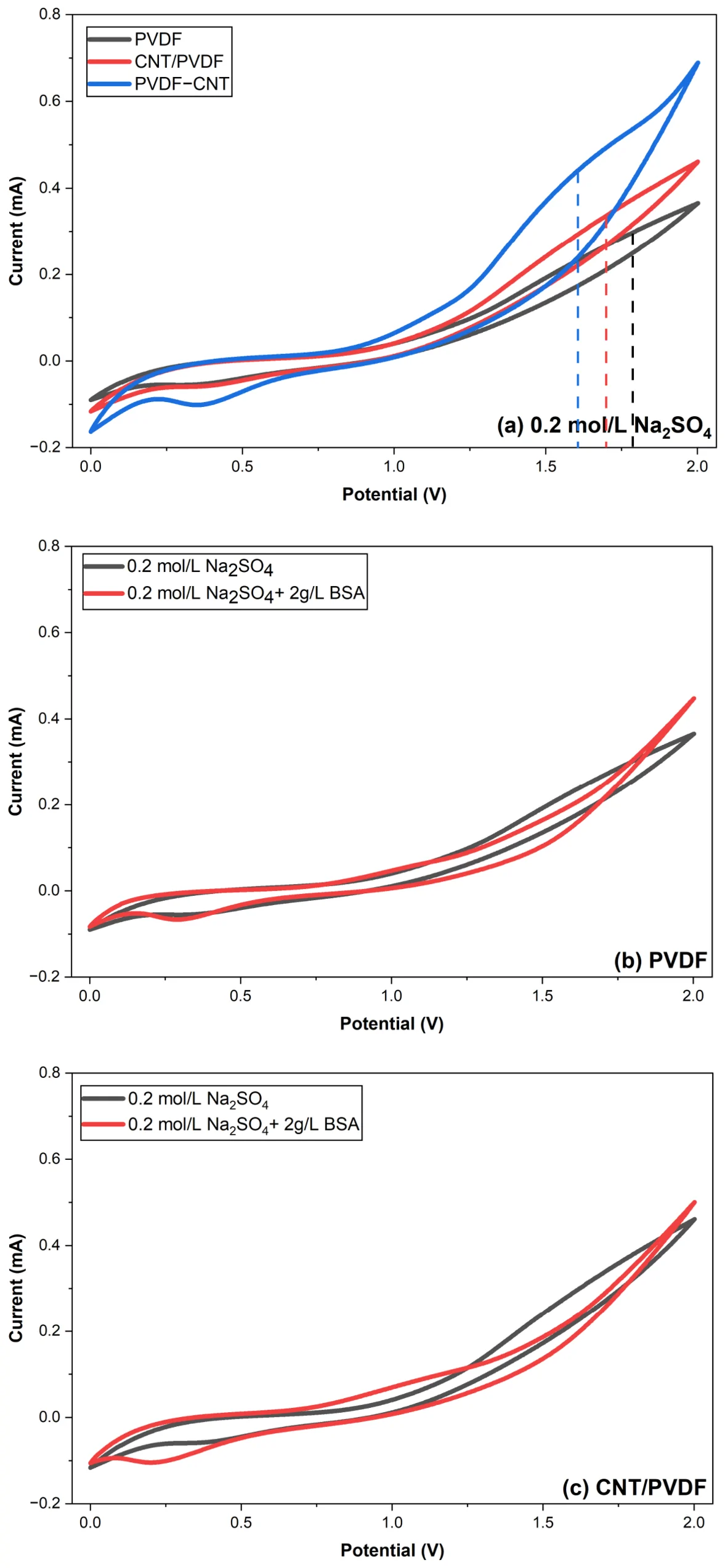

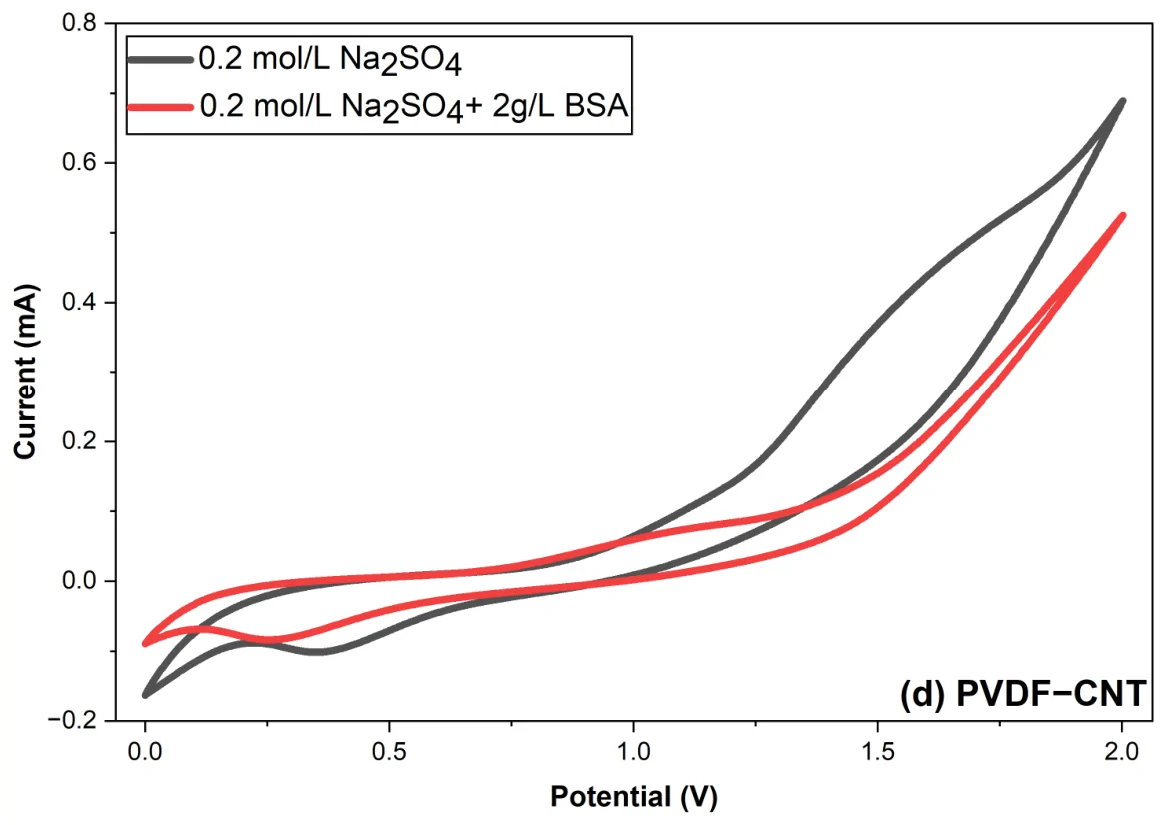
CV curves of pristine PVDF, CNT/PVDF and PVDF-CNT membranes in 0.2 mol/L Na_2_SO_4_ solution (**a**); CV curves of pristine PVDF membrane in 0.2 mol/L Na_2_SO_4_ solution with and without 2 g/L BSA (**b**); CV curves of CNT/PVDF membrane in 0.2 mol/L Na_2_SO_4_ solution with and without 2 g/L BSA (**c**); CV curves of PVDF-CNT membrane in 0.2 mol/L Na_2_SO_4_ solution with and without 2 g/L BSA (**d**).

In the CV curves shown in Figure 6a, outstanding oxidation peaks were observed ranging from +1.25 V to +1.8 V in the forward scan (from negative to positive) of the pristine PVDF, CNT/PVDF, and PVDF-CNT membranes. The CV peak of the PVDF-CNT membrane was systematically stronger and appeared at a lower potential than the other two counterparts. The peak potential of the PVDF-CNT membrane was around +1.6 V, which was lower than that of the PVDF (+1.8 V) and CNT/PVDF (+1.7 V) membranes, indicating that the PVDF-CNT membrane exhibited a more favourable threshold for the reaction to activate.

A clear difference in wet-state electrical resistance was identified, with the CNT/PVDF membrane exhibiting approximately half the resistance of the PVDF–CNT membrane (CNT/PVDF:PVDF-CNT = 1:2, Supplementary Materials Table S1 and Figure S3), since the CNTs were directly exposed to the electrolyte for CNT/PVDF membrane, while for the PVDF-CNT membrane, CNTs were incorporated into PVDF and indirectly exposed to the electrolyte. Although the PVDF-CNT membrane exhibited higher electrical resistance, its current at +1.6 V was still 1.5 times that of the CNT/PVDF membrane (Table 2). Therefore, the pronounced current response of the PVDF-CNT membrane at around +1.6 V could not be attributed to its electrical conductivity.

The intensified electrochemical response of the PVDF–CNT membrane at around +1.6 V was more likely associated with the polarisation-related behaviour of the PVDF-CNT membrane. The incorporation of CNTs promoted the formation of polar β-phase PVDF (Figure 4), thereby enhancing dipolar polarisation through the increased contribution of aligned molecular dipoles, while the heterogeneous PVDF–CNT interfaces, arising from the large contrast in their electrical properties, promoted local charge redistribution and accumulation, giving rise to enhanced interfacial polarisation [29]. Together, the enhanced dipolar and interfacial polarisation might contribute to a stronger overall polarisation response of the membrane [30]. However, the polarisation effects existed in a wide potential range, and there was a notable peak at +1.6 V (Figure 6d, black curves). When the potential approached +1.6 V, certain processes—such as surface reactions at CNT defect sites, redox activity of oxygen-containing groups, ion redistribution, or pseudocapacitive behaviour—might become activated, leading to a pronounced current peak. This response might be further synergistically amplified by the polarisation (interfacial polarisation, β-phase PVDF) [31,32]. At higher potentials, the peak became less distinguishable due to possible side reactions.

As shown in Figure 6b,c, there were insignificant differences for the CV curves of PVDF in electrolyte solution with and without BSA, suggesting that the pristine PVDF and CNT/PVDF membranes possessed weak, even negligible electrochemical activity in interacting with BSA. In contrast, the peak current of the PVDF-CNT membrane in the dilute BSA solution was distinctly different from the peak current measured in electrolyte solution without BSA (Figure 6d). After adding BSA to the electrolyte, strong adsorption of BSA onto the PVDF-CNT surface (via hydrophobic and π–π interactions) formed a non-conductive protein layer [33] and negatively affected the improved electrochemical response at the interface [34]. Consequently, the PVDF–CNT membrane exhibited a remarkable decrease in electrochemical response, leading to visibly smaller CV peaks. In contrast, the CV response of the pristine PVDF membrane remained largely unchanged after BSA addition, because BSA adsorption occurred on an intrinsically poorly conductive surface and therefore had only a limited effect on the overall electrochemical response (Figure 6b). From Figure 6c, the CNT/PVDF membrane exhibited a CV response similar to that of pristine PVDF, although slightly higher peak currents were observed due to the presence of the conductive CNT network. The limited change following BSA addition might be attributed to partial blockage of the exposed CNT sites, while the remaining interconnected CNT network continued to provide effective conductive pathways.

**Figure 7:**
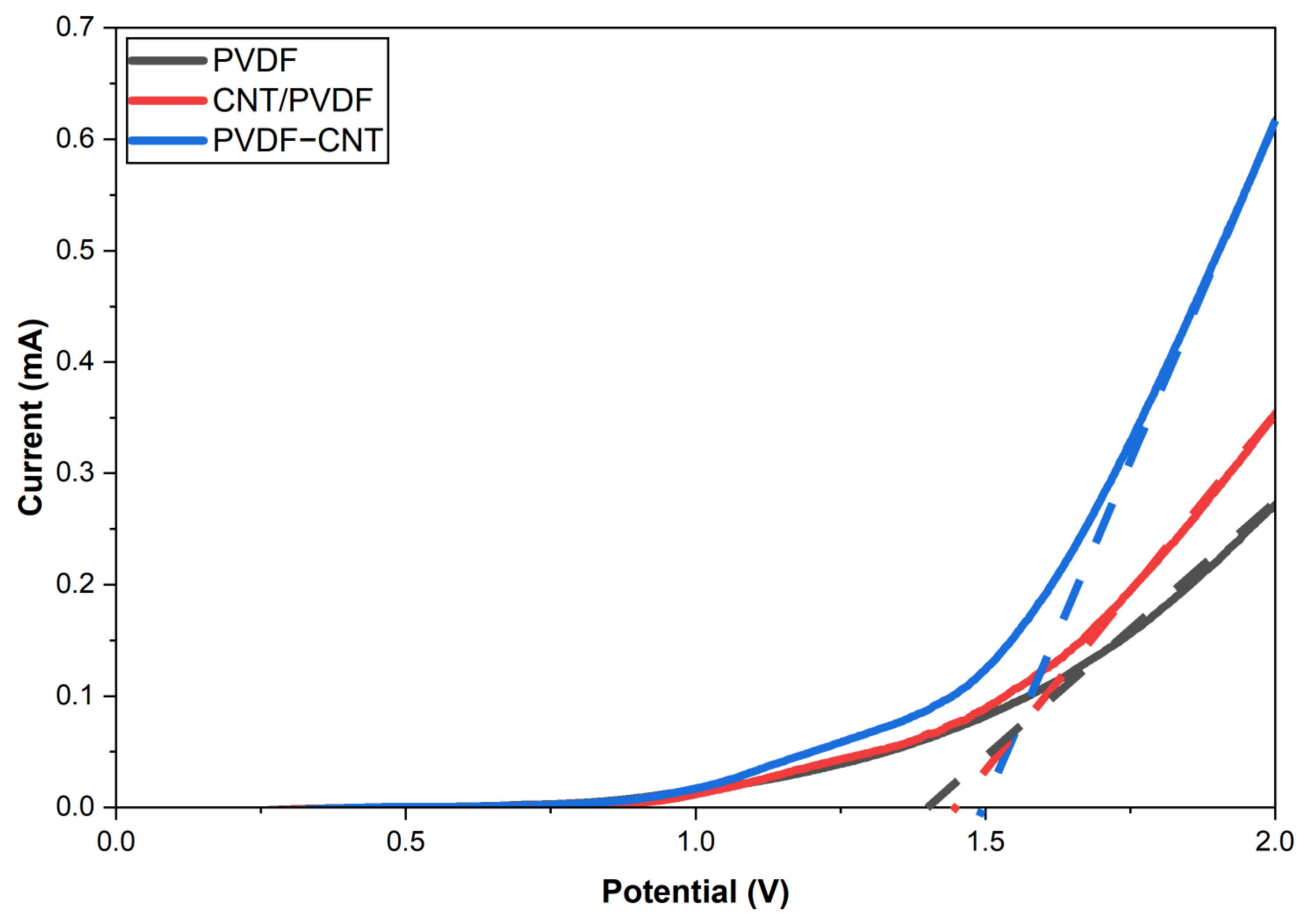
The LSV of pristine PVDF, CNT/PVDF and PVDF-CNT membranes in 0.2 mol/L Na_2_SO_4_ solution.

The OEP was determined from LSV curves by identifying the onset of the rapid anodic current increase (Figure 7). OEP of PVDF-CNT was remarkably increased, which meant that a lower side reaction of oxygen evolution occurred and a higher current efficiency was obtained at around +1.6 V [35].

**Figure 8:**
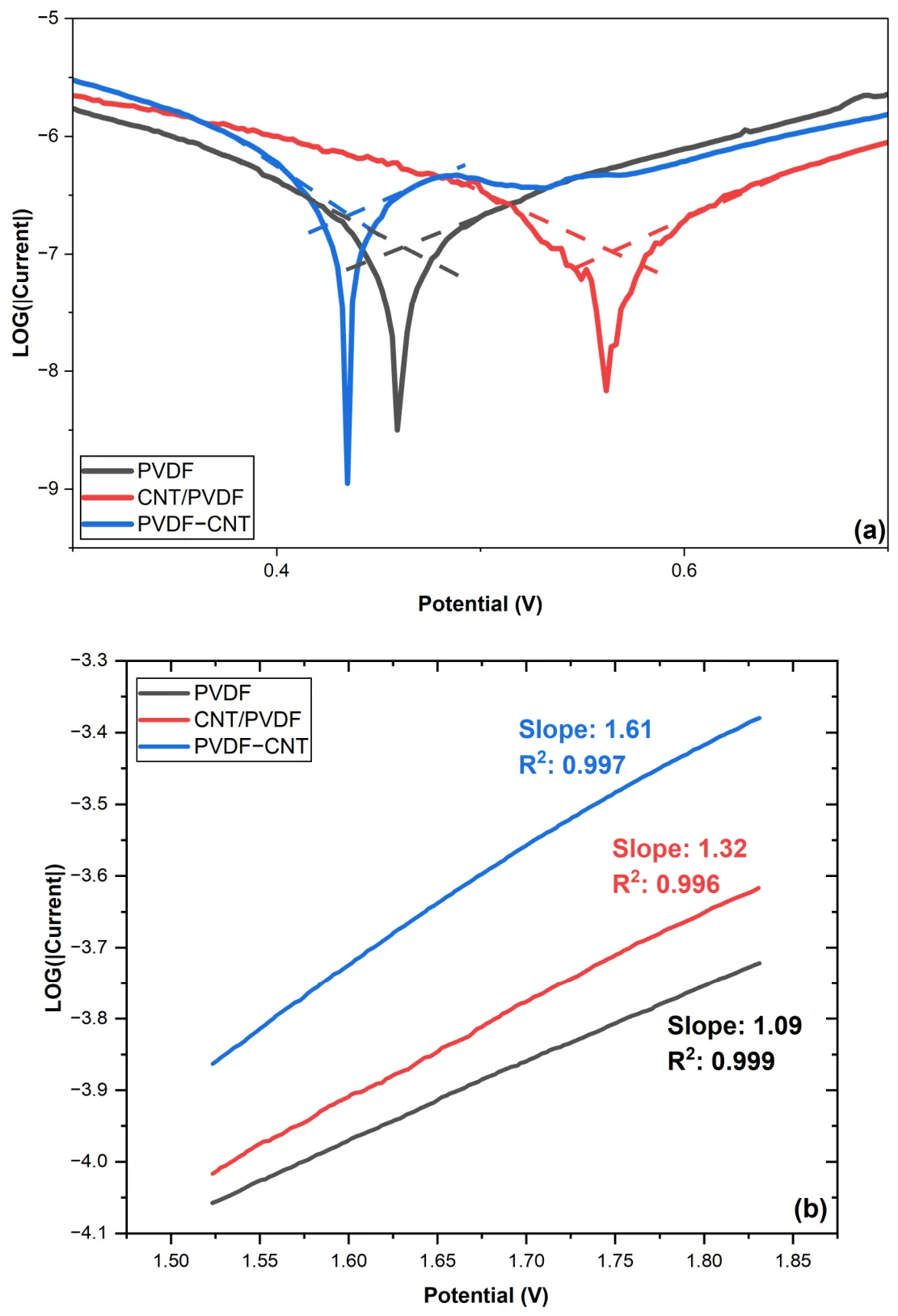
The Butler-Volmer plot (**a**) and kinetic slopes (**b**) of pristine PVDF, CNT/PVDF and PVDF-CNT membranes in 0.2 mol/L Na_2_SO_4_ solution.

The PVDF-CNT membrane (2E-7A) exhibited a higher apparent exchange current than the pristine PVDF (1E-7A) and CNT/PVDF membrane (1.1E-7A), indicating a more effective conductive network and enhanced intrinsic electrochemical activity, which was beneficial for electrochemical foulant removal. Furthermore, the higher gradients of the kinetic slope in the +1.6 V region of the PVDF-CNT membrane than those of the pristine PVDF and CNT/PVDF membranes (Figure 8b) suggested that the membrane possessed stronger electrochemical activity at around +1.6 V and could use lower potentials to achieve higher currents.

The enhanced electrochemical response and sensitivity to BSA adsorption of the PVDF-CNT membrane suggested +1.6 V as a candidate potential for investigating electrochemical BSA removal.

### 3.3. Membrane Filtration

**Figure 9:**
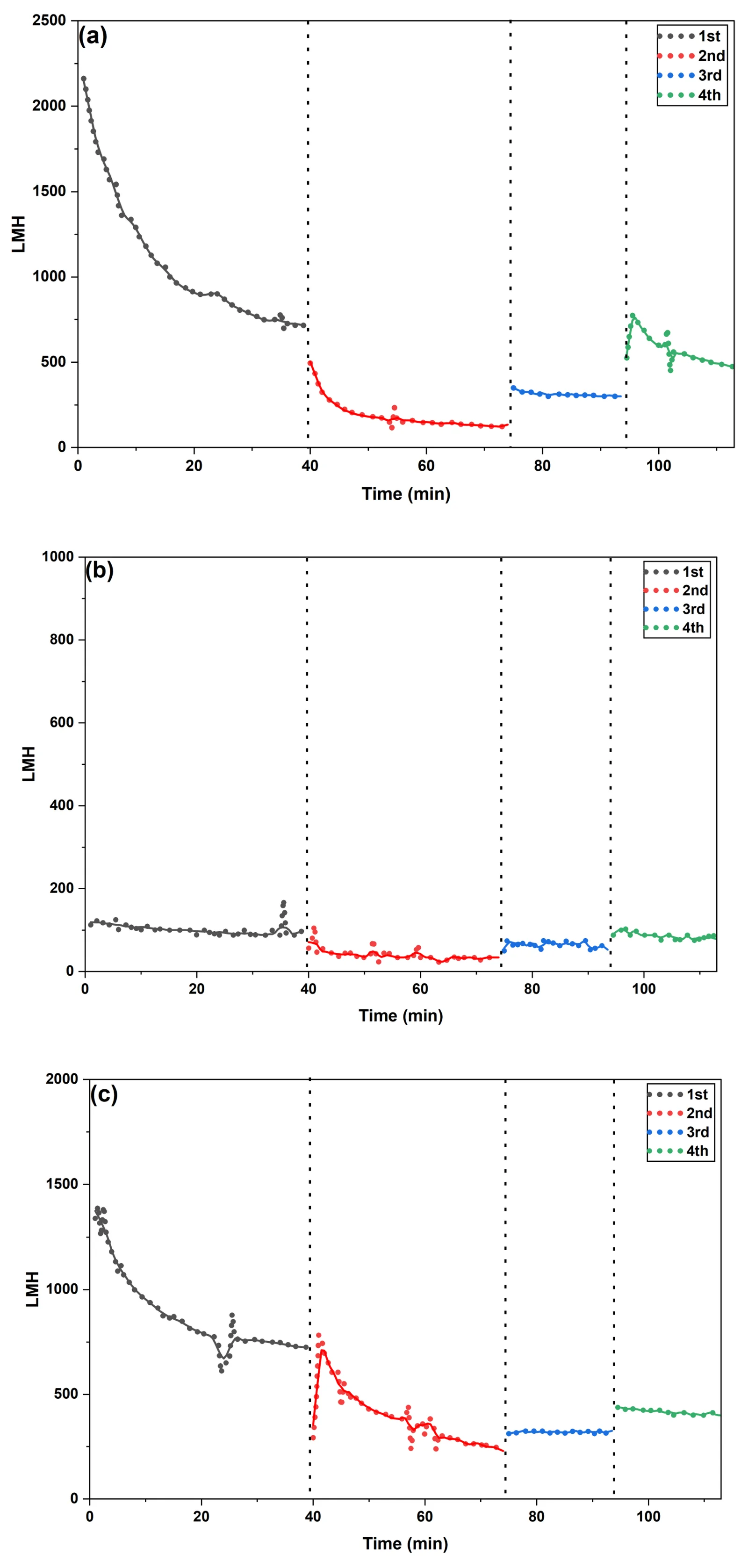
Filtration performance of PVDF-CNT (**a**), CNT/PVDF (**b**) and PVDF (**c**) membranes, rinsing with +1.6 V in 0.2 mol/L Na2SO4 (Stage 1: PW flux of clean membrane; Stage 2: BSA contamination; Stage 3: PW flux after contamination by BSA; Stage 4: PW flux after rinsing. The rinsing took place between Stage 3 and Stage 4.). The dots represent the measured data, while the solid lines represent the smoothed data.

As shown in Figure 9, the PVDF–CNT membrane exhibited higher initial PW flux than the pristine PVDF membrane (Stage 1), increasing from around 1400 LMH for PVDF to around 2250 LMH for PVDF–CNT. This improvement could be attributed to the increased membrane porosity induced by CNT incorporation [36]. However, during BSA filtration (Stage 2), the flux of the PVDF–CNT membrane became lower than that of PVDF. This behaviour resulted from the hydrophobic nature of CNTs, which could promote interactions with BSA molecules and thus aggravate membrane fouling [37]. Interestingly, the measured surface wettability of PVDF and PVDF–CNT membranes was nearly identical (Figure 1). This suggested that the difference in fouling behaviour was not mainly governed by the membrane surface wettability. Instead, it was likely that CNTs exposed on the pore surface increased the local hydrophobicity inside the membrane pores, facilitating BSA adsorption and pore blocking during filtration.

However, after electrochemically rinsing at +1.6 V in 0.2 mol/L Na_2_SO_4_ solution for 3 min, the PW flux of the PVDF-CNT membrane could bounce back to that of a fresh membrane at the end of the initial stage and maintained a much higher flux in PW filtration than that of the PVDF membrane (Stage 4). The FRR and R_C_ of the PVDF-CNT membrane achieved higher values (67% and 25%), compared to the PVDF counterpart (55% and 10%) (Table 3). Based on the electrochemical analysis characterisations, the improved ratios were mainly caused by CNT doping that boosted the removal of BSA foulant during the electrochemical rinsing process.

**Figure 10:**
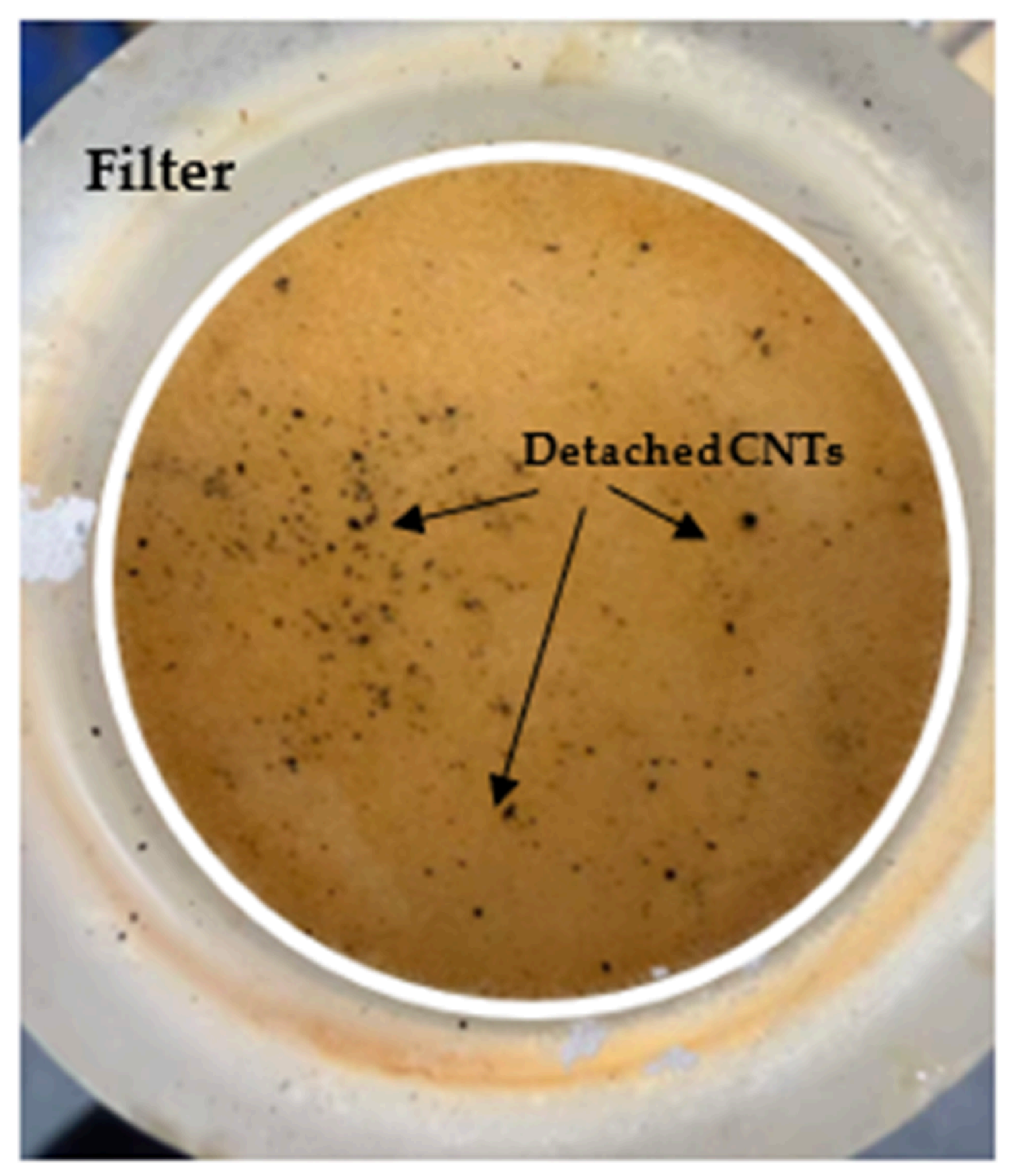
The detached CNTs from the CNT/PVDF membrane. The area confined in the white circle is the effective filtration area (9.6 cm^2^).

Although the CNT/PVDF membrane exhibited a very low flux compared with the other membranes, it showed the highest flux recovery ratio (FRR: 91%) after electrochemical rinsing at +1.6 V. This high FRR should be interpreted with caution. The vacuum-filtered CNT layer partially blocked the membrane pores, resulting in a suppressed initial flux and a limited effective filtration area. During electrochemical rinsing, the applied anodic potential and shear force likely induced detachment or degradation of loosely bound CNT aggregates, temporarily reopening some blocked pores (Figure 10). However, this recovery behaviour did not reflect effective BSA removal or stable electrochemical rinsing but rather indicated structural instability of the surface-coated CNT layer and potential CNT leakage under electrochemical conditions. Therefore, despite its high FRR, the CNT/PVDF membrane exhibited inferior practical performance compared with the PVDF-CNT membrane.

**Figure 11:**
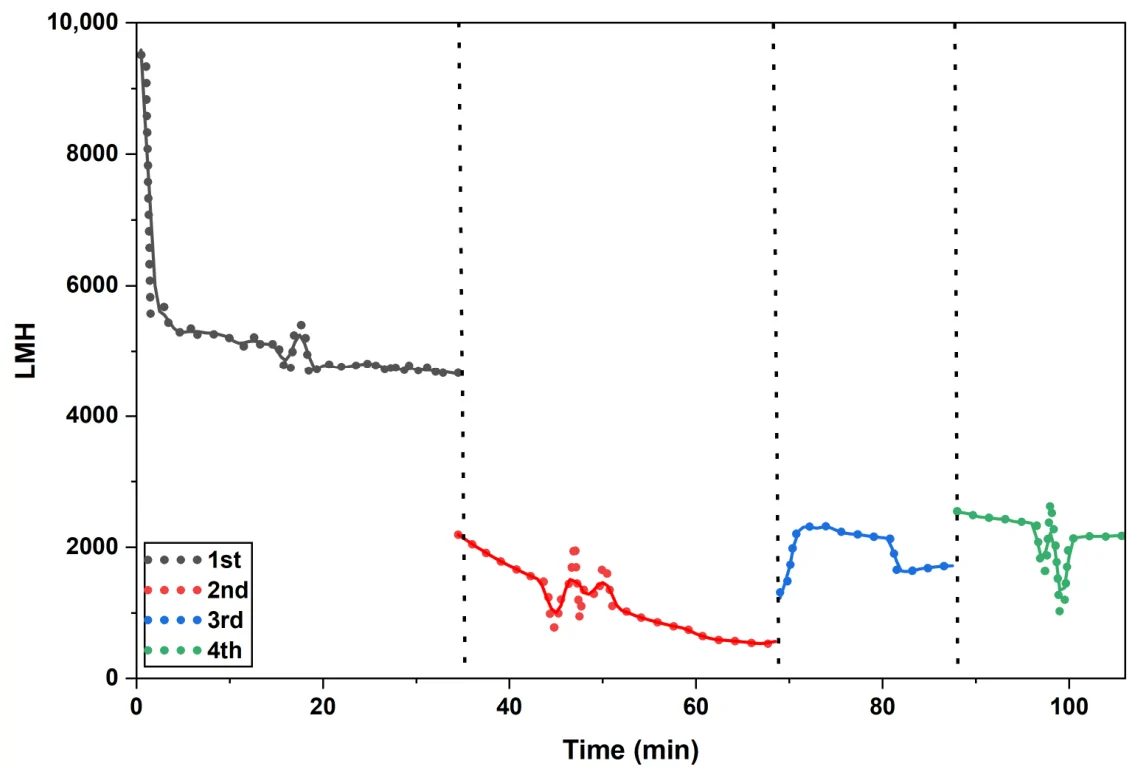
Filtration performance of the c-PVDF membrane, rinsed at +1.6 V in 0.2 mol/L Na_2_SO_4_ (Stage 1: PW flux of clean membrane; Stage 2: BSA contamination; Stage 3: PW flux after contamination by BSA; Stage 4: PW flux after rinsing. The rinsing took place between Stage 3 and Stage 4). The dots represent the measured data, while the solid lines represent the smoothed data. The Stage 1 for c-PVDF was 3-4 min shorter than lab made samples, but the PW flux had already reached the stable plateau. A few anomalously high or low flux values were observed, due to measurement noise from the electronic balance.

The c-PVDF membrane had a pore size of 0.1 µm, approximately ten times that of PVDF–CNT, and exhibited around 5 and 6 fold higher PW and BSA solution fluxes in the first and second filtration stages, respectively. Its hydrophilic surface modification promoted water adsorption on the surface and within pores, facilitating pore wetting and water transport. A hydration layer formed through hydrogen bonding could also partially shield the surface and reduce organic foulant adsorption. [38]. Nevertheless, despite this hydrophilic modification, the permeate flux of the c-PVDF membrane still declined under continuous BSA contamination and did not recover to an ideal stage (FRR: 46%) after using +1.6 V rinsing, indicating that hydrophilicity alone was insufficient to fully mitigate BSA fouling (Figure 11). Effective flux recovery (67%) was achieved for the PVDF-CNT membrane through electrochemical rinsing, leading to superior flux restoration compared with the c-PVDF membrane.

To further substantiate the contribution of electrochemical rinsing to BSA removal from the PVDF-CNT membrane, rinsing at +1.6 V was compared with a control sample performed without an applied potential, while all other experimental conditions were kept the same. The PVDF-CNT membrane rinsed at +1.6 V exhibited higher FRR (67% vs 33%) and R_C_ (25% vs 14%) than the sample rinsed without external potential (Supplementary Materials Figure S4 and Table S2).

**Figure 12:**
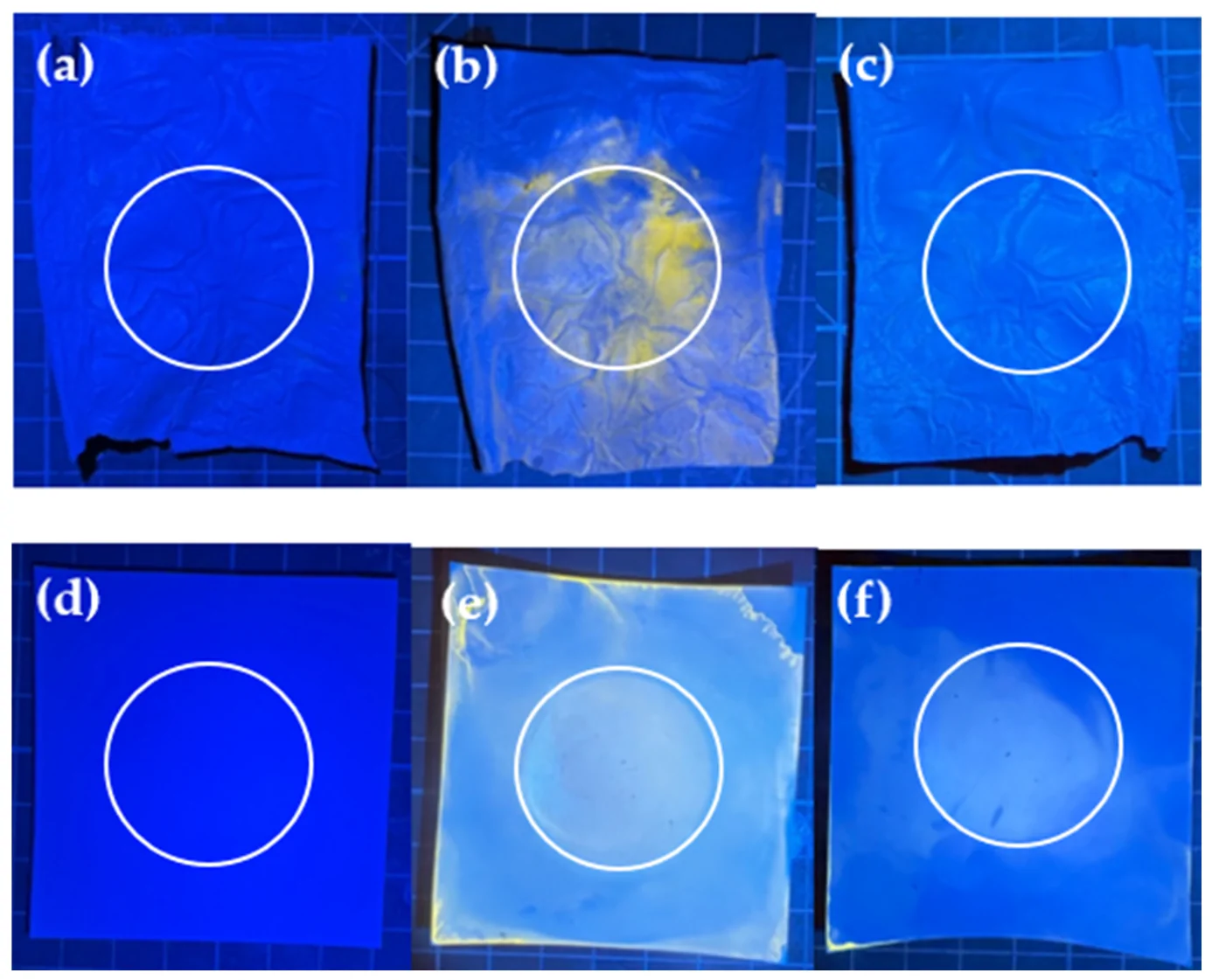
The clean (**a**), fouled (**b**) and electrochemically rinsed (**c**) PVDF-CNT membrane; The clean (**d**), fouled (**e**) and electrochemically rinsed (**f**) c-PVDF membrane. The white circle marked in the figures indicates the area used for filtration, namely the effective filtration area (9.6 cm^2^).

For the PVDF-CNT membrane, the fluorescence signal became nearly invisible after electrochemical rinsing, indicating effective removal of most of the adsorbed BSA (Figure 12c, within the white circle). In contrast, the c-PVDF membrane still showed obvious residual fluorescence after rinsing, suggesting more persistent BSA fouling (Figure 12f, within the white circle). Compared with the c-PVDF membrane, where foulant was more uniformly distributed across the effective filtration area (Figure 12e, within the white circle), the lab-made PVDF-CNT membrane exhibited a more uneven fouling pattern (Figure 12b, within the white circle), likely due to its local wrinkles and topographical variations, with BSA preferentially accumulating in certain regions while remaining weak or barely visible in others.

**Figure 13:**
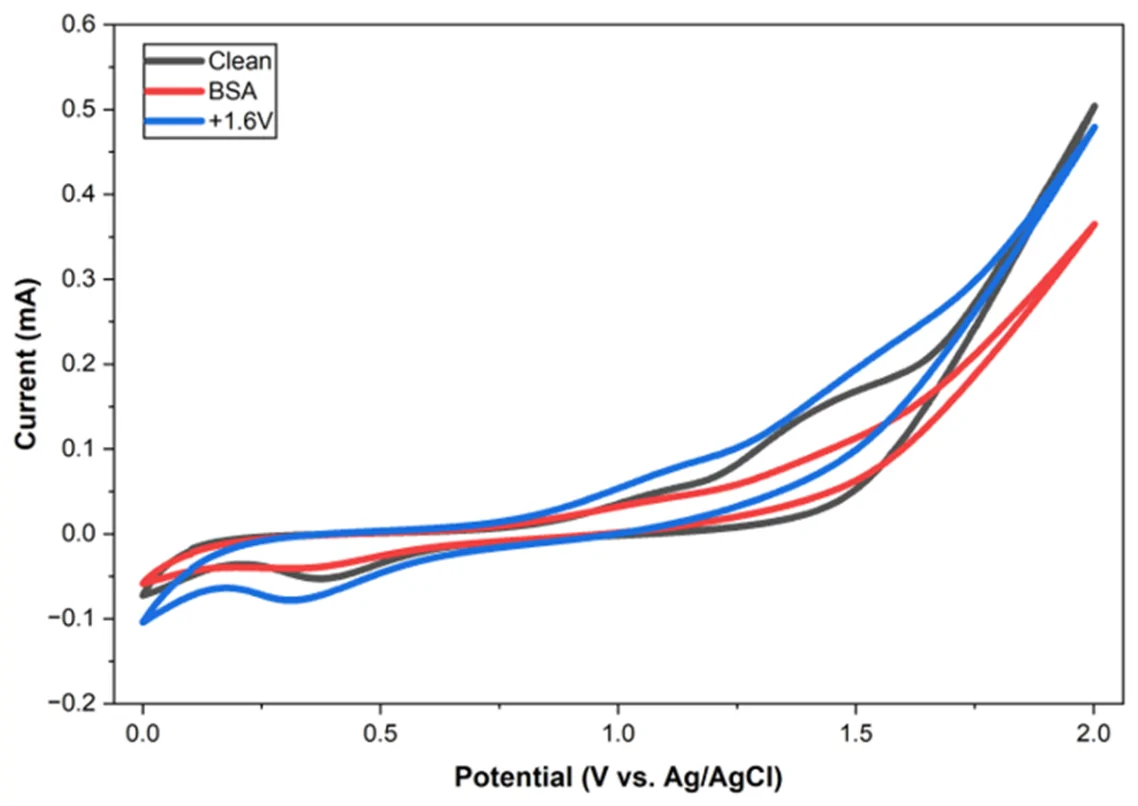
CV curves of clean, fouled and electrochemically rinsed PVDF-CNT membrane.

There were remarkable differences between the BSA-fouled and clean PVDF-CNT membranes in their anodic branch (Figure 13). The clean membrane showed increasing anodic current with potential and a peak near +1.6 V, whereas BSA fouling suppressed the current at +1–2 V, likely due to blockage of electroactive CNT sites by the adsorbed protein layer. After anodic rinsing at +1.6 V, the membrane showed markedly higher anodic current than the clean membrane over +0.8–1.8 V, although the peak near +1.6 V did not clearly recover. This phenomenon suggested that anodic rinsing at +1.6 V removed or oxidised bound BSA. During this anodic conditioning, highly reactive defect sites of CNT might be partially oxidised or passivated, while oxygen-containing functional groups (e.g., hydroxyl and carboxyl groups) were generated and distributed more uniformly across the CNT network [39]. As a result, electrolyte wettability and ion access were improved, allowing the electrolyte to reach previously less accessible CNT domains and increasing the effective electroactive area [40]. Consequently, the disappearance of the sharp anodic current peak together with the increased global current response reflected a transition from localised, heterogeneous surface reactions to more uniformly distributed electrochemical activity. This result demonstrated that although BSA adsorption temporarily suppresses CNT electroactivity, anodic treatment at +1.6 V effectively restored the electrochemical interface of the PVDF–CNT membrane.

**Figure 14:**
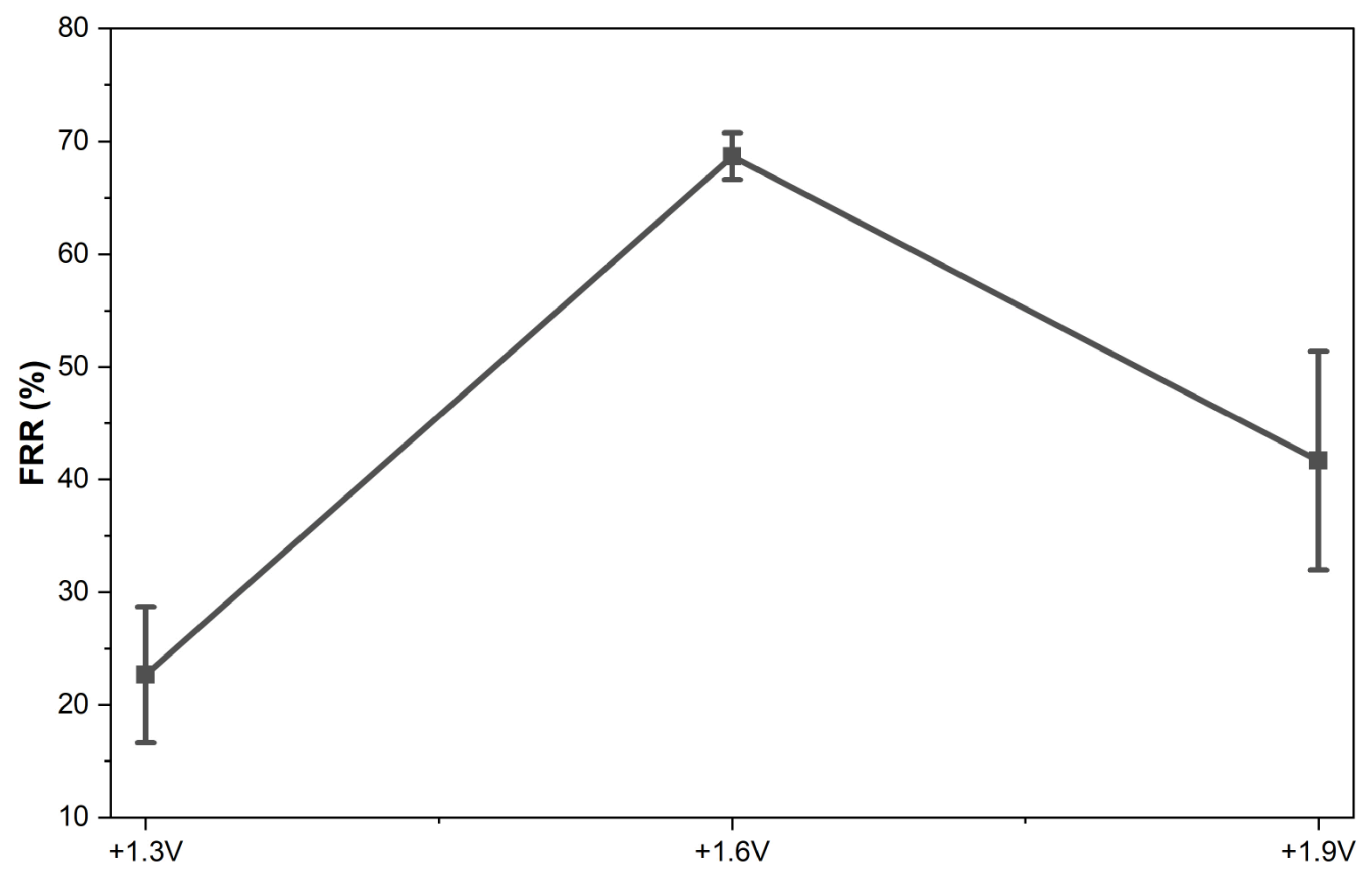
The FRR of the PVDF-CNT membrane after using +1.3 V, +1.6 V, and +1.9 V rinsing (Sample numbers: n = 3).

Among the tested potentials, +1.6 V resulted in the highest FRR (Figure 14), significantly outperforming rinsing at +1.3 V and +1.9 V. This optimal recovery at +1.6 V could be attributed to the balance between the membrane’s electrochemical activity, possible surface degradation and side reactions at higher potentials (e.g., +1.9 V). The combined CV (Figure 6) and FRR results (Figure 14) demonstrated that +1.6 V represented an optimal rinsing potential at the CV peak region, where sufficient anodic electrochemical activity was achieved to remove BSA efficiently.

### 3.4. Stability

**Figure 15:**
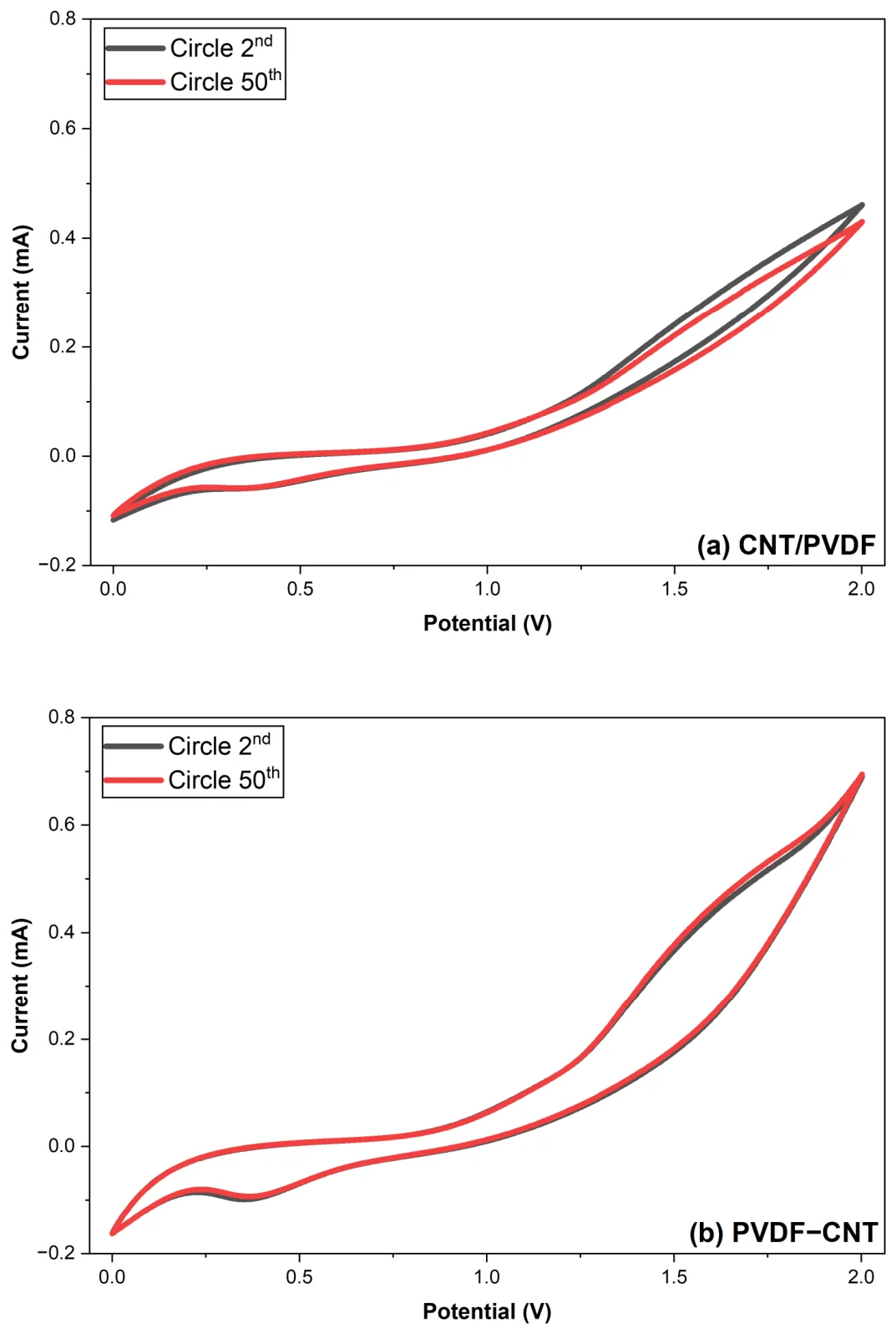
CVs of CNT/PVDF (**a**) and PVDF-CNT (**b**) membranes for the electrochemical rinsing stability test.

The PVDF-CNT membrane exhibited significantly higher stability than the CNT/PVDF membrane during the electrochemical scanning process. The current value around +1.6 V for the CNT/PVDF membrane gradually decreased as the number of scans increased from 2 to 50, while the CV signal in the same potential range for the PVDF-CNT membrane remained almost the same level (Figure 15). In the incorporated membrane, CNTs were embedded within the PVDF matrix, providing strong bonding and preventing CNT detachment. In contrast, the surface-coated membrane relied mainly on weak physical adhesion between CNTs and the PVDF surface, leading to partial CNT loss or surface damage. This demonstrated that incorporating CNTs into the membrane structure effectively enhanced electrochemical stability and durability.

Besides the electrochemical stress during the rinsing, the membranes were also subjected to the rinsing shear force, as well as the vertical water flux force during the filtration. As shown in Figure 10, significant CNT leakage from the CNT surface-coated membrane was observed when subjected to shear forces during rinsing and the vertical hydraulic force during water-flux filtration, indicating evident unsatisfactory stability during filtration. Therefore, the filtration stability test was only conducted for the c-PVDF and PVDF-CNT membranes.

**Figure 16:**
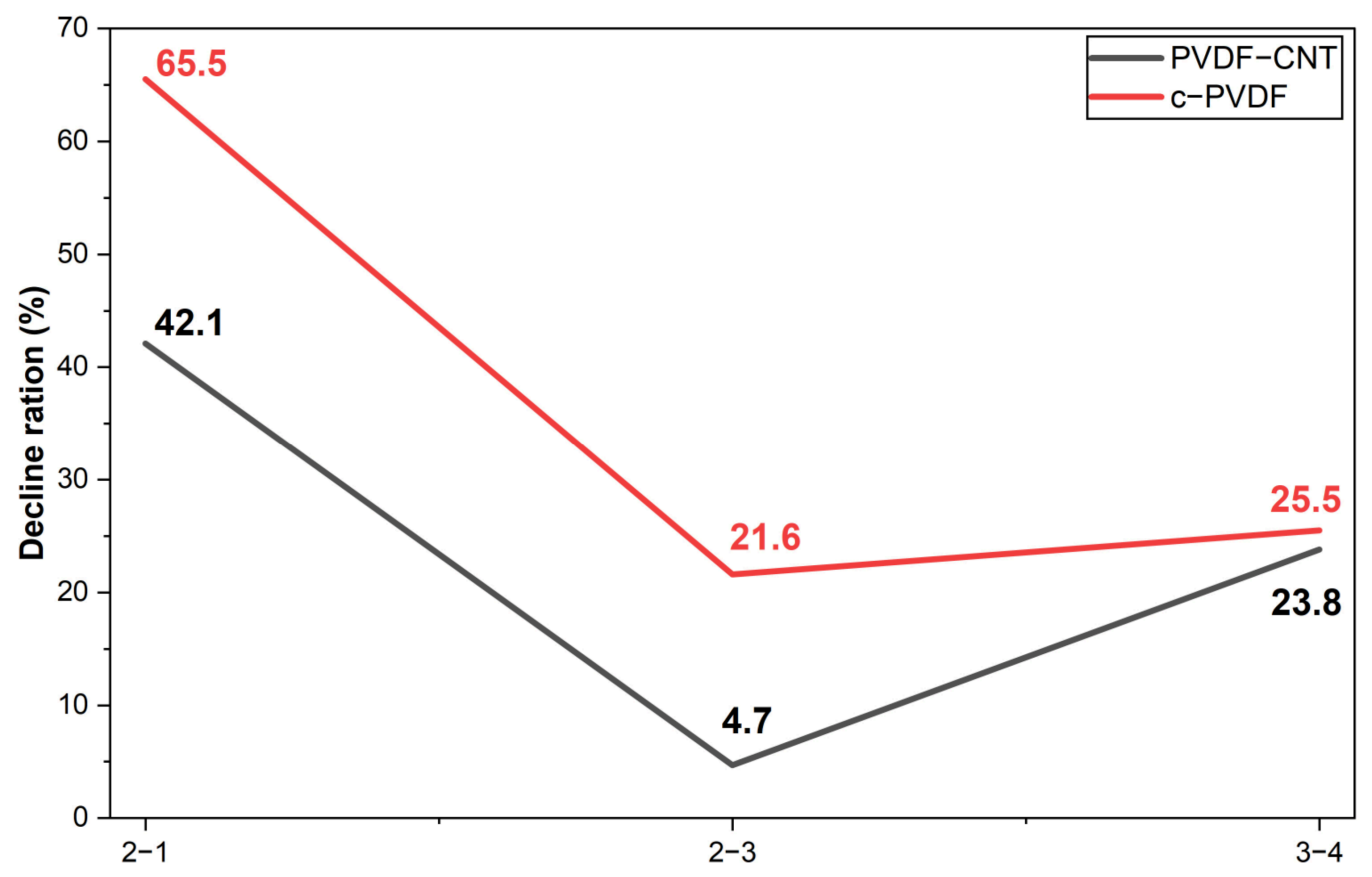
The FRR changes for PVDF-CNT and c-PVDF membranes (2-1 indicates the decline in recovery rate between the 2nd circle and the 1st circle). The flux changes for c-PVDF and PVDF-CNT membranes after 4 BSA contaminations followed by electrochemical rinsing were shown in Supplementary Materials Figure S5.

Figure 16 showed the FRR changes in c-PVDF and PVDF–CNT over four successive BSA contamination–electrochemical rinsing cycles. Both membranes experienced FRR loss, but conductive PVDF–CNT generally exhibited smaller declines than non-conductive c-PVDF. The FRR decline ratios for PVDF–CNT and c-PVDF were 42.1% and 65.5%, respectively, in the first comparison (2-1), decreasing to 4.7% and 21.6% in the second (2-3). In the final comparison (3-4), these ratios increased to 23.8% and 25.5%, respectively, narrowing the difference between the membranes. These results indicate better relative flux retention by PVDF–CNT under the tested conditions, potentially associated with its electrochemical interaction with BSA at +1.6 V. However, the substantial final decline shows that electrochemical rinsing did not fully prevent fouling-related flux loss during repeated cycles.

## 4. Conclusions

In summary, pristine PVDF and PVDF–CNT membranes were fabricated by phase inversion, while CNT/PVDF membranes were prepared by vacuum filtration. SEM, Raman spectroscopy, XRD, and EDS confirmed the successful CNT incorporation and surface coating. CNT incorporation into PVDF could induce the formation of β-phase PVDF. At approximately +1.6 V, PVDF–CNT exhibited the highest peak current (0.44 mA), compared with CNT/PVDF (0.28 mA) and pristine PVDF (0.23 mA). This enhanced response might be induced by the combined contributions of β-phase-associated dipolar polarisation and interfacial polarisation, potentially promoting charge accumulation and redistribution. After BSA addition, the PVDF–CNT exhibited the most sensitive response to BSA interference at +1.6 V. Accordingly, +1.6 V was selected for subsequent electrochemical rinsing. In dead-end filtration tests, PVDF–CNT achieved a FRR of 67% following electrochemical rinsing, exceeding those of pristine PVDF (55%) and c-PVDF (47%). The higher apparent recovery of CNT/PVDF (91%) was attributed to CNT detachment and pore reopening, indicating limited coating stability. Comparative CV analyses of fresh, fouled, and electrochemically rinsed PVDF–CNT membranes, together with FITC-BSA imaging of PVDF–CNT and c-PVDF as well as stability tests, further supported the electrochemical removal of BSA foulant and associated flux recovery. These findings demonstrated the structural stability and electrochemical rinsing performance of PVDF-CNT membranes and suggested that tailoring polarisation-related membrane behaviour provided a direction for improving electrochemically assisted rinsing in water treatment. Nevertheless, further cross-sectional imaging and pore-size distribution analysis would provide additional insight into how the internal membrane structure contributes to the observed filtration and electrochemical rinsing performance.

## Figures and Tables

**Table 1:** The amount of materials for the casting solution.

Component	PVDF (g)	PVDF-CNT (g)
**PVDF**	1.5	1.5
**PVP**	0.1	0.1
**NMP**	8.3	8.3
**CNT**	N/A	0.0375
**EtOH**	0.1	0.1
**Total casting solution mass**	10	10.0375

**Table 2:** The current value at +1.6 V estimated from Figure 6.

Samples	I_Peak (mA)	I_Peak_BSA (mA)
**PVDF**	0.23	0.19
**CNT/PVDF**	0.28	0.23
**PVDF-CNT**	0.44	0.2

**Note:** I_p represents peak current; I_p_BSA represents peak current when BSA was added to the electrolyte.

**Table 3:** The FRR, R_t_, R_C_ and flux data used to calculate these ratios.

Samples	J_w0_ (LMH)	J_w1_ (LMH)	J_w2_ (LMH)	FRR	R_t_	R_C_
**PVDF**	725	325	400	55%	58%	10%
**CNT/PVDF**	87.5	56.25	80	91%	36%	27%
**PVDF-CNT**	712.5	300	475	67%	58%	25%
**c-PVDF**	4662.5	1712.5	2175	47%	63%	10%

## Data Availability

The original contributions presented in this study are included in the article/Supplementary Material. Further inquiries can be directed to the corresponding author.

## Supplementary Materials

The following supporting information can be downloaded at https://www.mdpi.com/article/10.3390/membranes16100351/s1: S1: Calculation of the CNT surface coating amount; Table S1: The EIS equivalent simulation results (R_s_ represented to the series resistance, R_m_ represented to the wet membrane resistance, C represented to the wet membrane capacitance, W referred to the diffusion impedance). The simulation frequency range was from 504660 to 0.1 Hz; Table S2: The FRR, R_t_ and R_C_ and flux data used to calculate the ratios; Figure S1: The dead-end filtration set up; Figure S2: The electrochemical rinsing set up (The red one connects with the membrane as the working electrode; the blue one connects with the reference electrode; the black one connects with the counter electrode); Figure S3: The EIS Nyquist plot and equivalent circuit; Figure S4: Filtration performance of PVDF-CNT membranes, rinsing by 0.2 mol/L Na_2_SO_4_ (Stage 1: PW flux of clean membrane; Stage 2: BSA contamination; Stage 3: PW flux after contaminated by BSA; Stage 4: PW flux after rinsing. The rinsing took place between Stage 3 and Stage 4.). The dots represent the measured data, while the solid lines represent the smoothed data. Figure S5: The filtration fluxes for c-PVDF and PVDF-CNT membranes after each fouling-electrochemical rinsing circle.

## Abbreviations

The following abbreviations are used in this manuscript:

PVDF	Polyvinylidene fluoride
CNT	Carbon-nanotube
Ethanol	EtOH
PVP	Polyvinylpyrrolidone
NMP	N-Methyl-2-pyrrolidone
NIPS	Non-solvent induced phase separation
FITC-BSA	Fluorescein isothiocyanate–conjugated bovine serum albumin
