# Supplementary material for: Electrochemical Removal of BSA Foulant Using a Stabilised Conductive PVDF Membrane Incorporated with Multiwall Carbon Nanotubes
Source: Membranes (Basel). 2026 Oct 21;16(10):351. doi: 10.3390/membranes16100351 (PMC13707198; doi:10.3390/membranes16100351)
Supplement: Supplementary file 1 [file membranes-16-00351-s001.zip › membranes-4609560-supplementary.pdf]

Supplementary Materials

S1. Calculation of the CNT surface coating amount:

Assuming that NMP and ethanol are removed during phase inversion, washing, and drying, and that the residual PVP content is negligible, the final dry membrane is considered to consist mainly of:

PVDF and CNT

Take one PVDF-CNT sample.

The total mass of PVDF and CNT is:

$$m_{PVDF+CNT} = 1.5 + 0.0375 = 1.5375 \text{ g}$$

The theoretical CNT mass fraction in this dry membrane is:

$$w_{CNT} = \frac{0.0375}{1.5375} = 0.02439$$

Therefore:

$$\boxed{w_{CNT} = 2.439 \text{ wt\%}}$$

Note that the original 2.5 wt% refers to:

$$\frac{m_{CNT}}{m_{PVDF}}$$

whereas 2.439 wt% refers to:

$$\frac{m_{CNT}}{m_{PVDF} + m_{CNT}}$$

The dry PVDF-CNT membrane sample weighs 0.11g.

Therefore, the theoretical CNT mass in this dry PVDF-CNT membrane sample is:

$$m_{CNT} = 0.11 \times 0.02439 = 0.0026829 \text{ g}$$

Thus:

$$\boxed{m_{CNT} = 2.6829 \text{ mg}}$$

The total membrane area for the PVDF-CNT sample which used to determine the CNT loading per unit membrane area is:

$$A_{total} = 37 \text{ cm}^2$$

Thus, for example, the CNT coating mass for the CNT surface coated PVDF membrane corresponding to the effective filtration area 9.6 cm^2 area is:

$$m_{CNT,9.6} = 9.6 \times (\frac{2.6829}{37} = 0.07251\ mg/cm^2) = 0.696mg$$

S2. The EIS equivalent simulation results

Table S1. The EIS equivalent simulation results (R_s represented to the series resistance, R_m represented to the wet membrane resistance, C represented to the wet membrane capacitance, W referred to the diffusion impedance). The simulation frequency range was from 504660 to 0.1 Hz.

	R_s	R_m	C	W	$X^2(Z)$	Convergence
**CNT/PVDF**	1.28 KΩ	29.7 KΩ	43.6 μF	135 μFho*s^(1/2)	5.22E-09	Yes
**PVDF-CNT**	664 Ω	55.7 KΩ	68.3 μF	152 μFho*s^(1/2)	5.19E-07	Yes

S3. Comparison of Flux Recovery after Cleaning of PVDF–CNT Membranes with and without an Applied Potential

Table S2. The FRR, R_t and R_C and flux data used to calculate the ratios.

Samples	J_{w0} (LMH)	J_{w1} (LMH)	J_{w2} (LMH)	FRR	R_t	R_C
**PVDF-CNT +1.6V 0.2 mol/L Na_2SO_4**	712.5	300	475	67%	58%	25%
**PVDF-CNT 0.2 mol/L Na_2SO_4**	825	156.25	275	33%	81%	14%

S4. The dead-end filtration set up

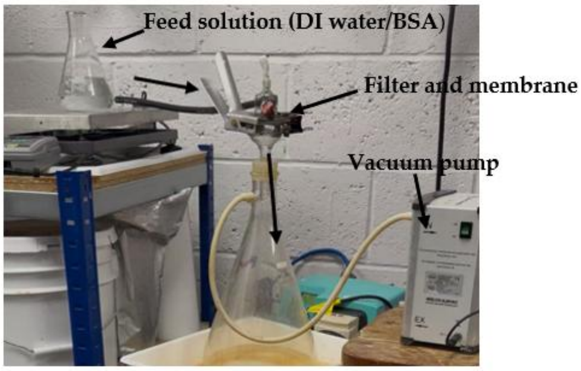

Figure S1. The dead-end filtration set up.

S5. The electrochemical cleaning set up

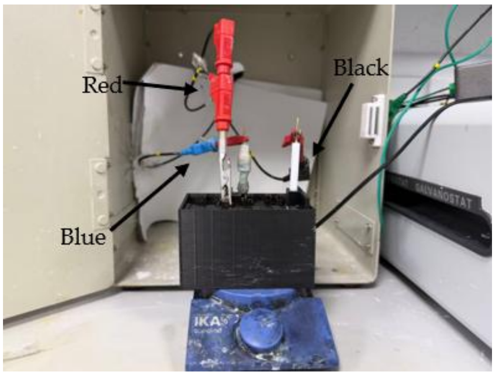

Figure S2. The electrochemical cleaning set up (The red one connects with the membrane as the working electrode; the blue one connects with the reference electrode; the black one connects with the counter electrode).

S6. The EIS results and equivalent circuit

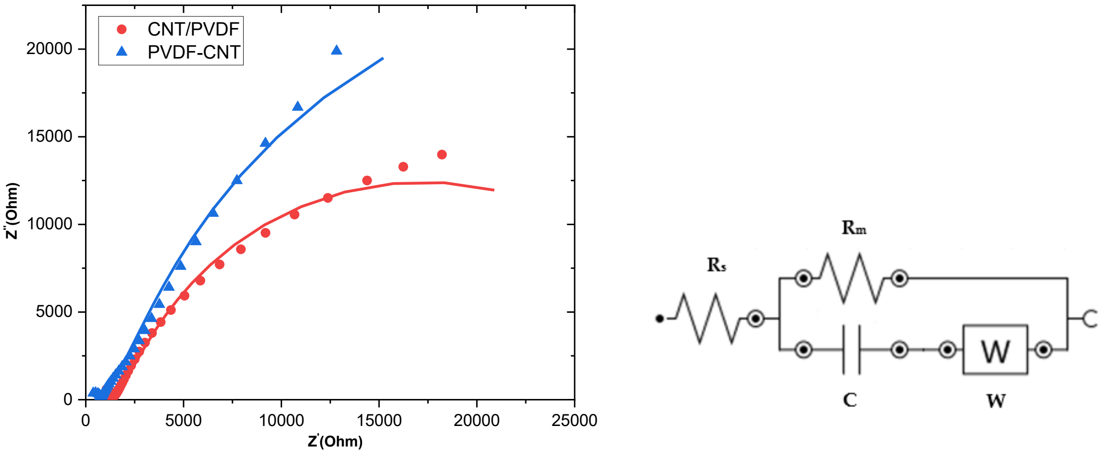

Figure S3. The EIS Nyquist plot and equivalent circuit.

Besides the resistance information obtained from the fitting results of equivalent circuit (**Supplementary Materials Table S1**), the higher capacitance of the PVDF–CNT membrane at OCP compared to that of CNT/PVDF membrane might be attributed to interfacial polarisation at the PVDF and CNT interfaces and the formation of polar β-phase PVDF, which together enhance the charge-storage capability of the membrane by facilitating charge redistribution and accumulation. This was also an additional evidence to support the polarisation related behaviour of PVDF-CNT membrane.

S7. PVDF-CNT filtration results rinsing without external potential

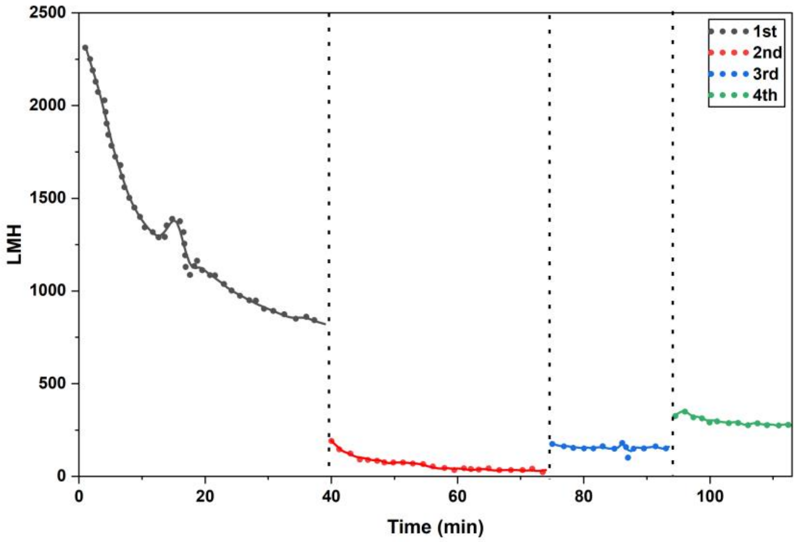

Figure S4. Filtration performance of PVDF-CNT membranes, rinsing in 0.2 mol/L Na_2SO_4 (Stage 1: PW flux of clean membrane; Stage 2: BSA contamination; Stage 3: PW flux after contamination by BSA; Stage 4: PW flux after rinsing. The rinsing took place between Stage 3 and Stage 4.). The dots represent the measured data, while the solid lines represent the smoothed data.

S8. Filtration flux changes after +1.6V rinsing for c-PVDF and PVDF-CNT membranes

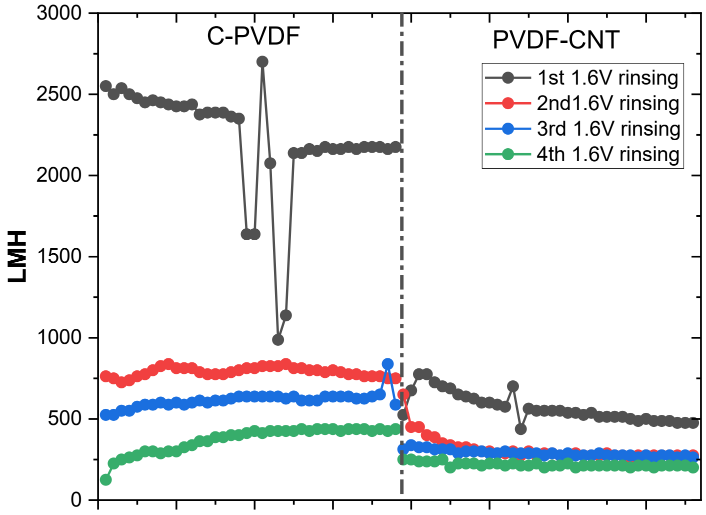

Figure S5. The filtration fluxes for c-PVDF and PVDF-CNT membranes after each fouling-electrochemical rinsing circle.
